# CLK1/SRSF5 pathway induces aberrant exon skipping of METTL14 and Cyclin L2 and promotes growth and metastasis of pancreatic cancer

**DOI:** 10.1186/s13045-021-01072-8

**Published:** 2021-04-13

**Authors:** Shi Chen, Can Yang, Zu-Wei Wang, Jian-Fei Hu, Jing-Jing Pan, Cheng-Yu Liao, Jia-Qiang Zhang, Jiang-Zhi Chen, Yi Huang, Long Huang, Qian Zhan, Yi-Feng Tian, Bai-Yong Shen, Yao-Dong Wang

**Affiliations:** 1 Shengli Clinical Medical College of Fujian Medical University, Fujian Medical University, No. 134, East Street, Fuzhou, 350001 Fujian Province People’s Republic of China; 2 Department of Hepatobiliary Pancreatic Surgery, Fujian Provincial Hospital, Fuzhou, 350001 People’s Republic of China; 3 Department of Hepatobiliary Surgery, Union Hospital, Fujian Medical University, Fuzhou, 350001 People’s Republic of China; 4 Department of General Surgery, Pancreatic Disease Center, Research Institute of Pancreatic Diseases, Ruijin Hospital, Shanghai Jiao Tong University School of Medicine, No.197 Ruijin Second Road, Shanghai, 200025 People’s Republic of China; 5 Center for Experimental Research in Clinical Medicine, Fujian Provincial Hospital, Fuzhou, 350001 People’s Republic of China

**Keywords:** Pancreatic cancer, Alternative splicing, CLK1, SRSF5, M6A Modification, METTL14, Cyclin L2

## Abstract

**Background:**

Both aberrant alternative splicing and m6A methylation play complicated roles in the development of pancreatic cancer (PC), while the relationship between these two RNA modifications remains unclear.

**Methods:**

RNA sequencing (RNA-seq) was performed using 15 pairs of pancreatic ductal adenocarcinoma (PDAC) tissues and corresponding normal tissues, and Cdc2-like kinases 1 (CLK1) was identified as a significantly upregulated alternative splicing related gene. Real-time quantitative PCR (qPCR) and western blotting were applied to determine the CLK1 levels. The prognostic value of CLK1 was elucidated by Immunohistochemistry (IHC) analyses in two independent PDAC cohorts. The functional characterizations and mechanistic insights of CLK1 in PDAC growth and metastasis were evaluated with PDAC cell lines and nude mice. SR-like splicing factors5^250-Ser^ (SRSF5^250-Ser^) was identified as an important target phosphorylation site by phosphorylation mass spectrometry. Through transcriptome sequencing, Methyltransferase-like 14^exon10^ (METTL14^exon10^) and Cyclin L2^exon6.3^ skipping were identified as key alternative splicing events regulated by the CLK1-SRSF5 axis. RIP assays, RNA-pulldown and CLIP-qPCR were performed to confirm molecular interactions and the precise binding sites. The roles of the shift of METTL14^exon 10^ and Cyclin L2^exon6.3^ skipping were surveyed.

**Results:**

CLK1 expression was significantly increased in PDAC tissues at both the mRNA and protein levels. High CLK1 expression was associated with poor prognosis. Elevated CLK1 expression promoted growth and metastasis of PC cells in vitro and in vivo. Mechanistically, CLK1 enhanced phosphorylation on SRSF5^250-Ser^, which inhibited METTL14^exon10^ skipping while promoted Cyclin L2^exon6.3^ skipping. In addition, aberrant METTL14^exon 10^ skipping enhanced the N6-methyladenosine modification level and metastasis, while aberrant Cyclin L2^exon6.3^ promoted proliferation of PDAC cells.

**Conclusions:**

The CLK1/SRSF5 pathway induces aberrant exon skipping of METTL14 and Cyclin L2, which promotes growth and metastasis and regulates m6A methylation of PDAC cells. This study suggests the potential prognostic value and therapeutic targeting of this pathway in PDAC patients.

**Supplementary Information:**

The online version contains supplementary material available at 10.1186/s13045-021-01072-8.

## Background

As the fourth most leading cause of cancer-related death in the world, pancreatic cancer (PC) is usually diagnosed at an advanced stage [1]. The rising mortality rates from PC has projected it will become the second common cause of tumor-related mortality by the year 2030 [2]. Five-year survival rate of PC is only 8% so far and most patients die within 7 years of surgery [3], indicating a very poor prognosis of PC patients. Although accumulating genetic studies of PC have identified a mass of alterations in crucial genes [4–6], the molecular mechanisms underlying the development of PC are still much more to be discovered. Therefore, there is a strong demand for finding effective therapeutic strategies that can facilitate early detection and improve the prognosis of PC, such as the common pancreatic ductal adenocarcinoma (PDAC).

Emerging as the most prevalent mechanisms of gene regulation, alternative splicing (AS) and N6-methyladenosine (m6A), have emerged as the prevalent gene modifications in human cancers [7–9]. AS has been shown to play a crucial role in the complicated regulation of protein functions and the dysregulation of splicing factors is closely associated with initiation and progression of human cancers [7]. So, splicing factors have been confirmed as oncogenic or suppressive drivers in multiple malignant tumors [10, 11]. It is predicted that more than 90% of transcripts from interethnic protein-coding transcripts could be spliced alternatively in a tissue, under stress or in disease [12]. While the average human gene just produces three or more alternatively spliced mRNA isoforms, the tumor cells produce a significant redundant of splice variants [13, 14]. These atypical splice variants reside at the core of human cancer development and progression. On the other hand, the roles of m6A in cancers have been increasingly explored in recent years [15–17]. m6A modification is a reversible process which is mediated by the m6A methyltransferases including methyltransferase-like 14 (METTL14), methyltransferase-like 3 (METTL3), and Wilms tumor 1 associated protein (WTAP) [18–22]. Since m6A modifications functions critically in homeostasis maintenance of cells, aberrant m6A modifications were widely reported to be associated with tumorgenesis and tumor development, in which process, the protein effects of “writer” METTL14 are significant. For example, decreased METTL14 mutation in endometrial cancer reduces the m6A modification of AKT pathway-related genes, resulting in the activation of the AKT signaling pathway and induction of tumorigenesis [23]. In addition, METTL14 up regulation causes a decrease in PERP expression through m6A modification, leading to PC growth and metastasis [24]. While, the function of alternative splicing of m6A modification related proteins including METTL14 has not been reported in the world. Thus, exploration of METTL14 splicing mediated m6A RNA methylation in tumorigenesis and cancer development may be potentially interesting.

Serine/arginine-rich splicing factors (SRs) belongs to the splicing factor category of RNA-binding proteins (RBPs) that regulate RNA splicing and other RNA-modification associated events [25]. Phosphorylation is crucial for both splicing and splicing-independent functions of SR proteins. Composed of classical SR-splicing factors (SRSFs) and RNA binding SR-like splicing factors [10], SR proteins are phosphorylated predominantly within the RS domain by two families of protein kinases, namely Cdc2-like kinases (CLKs) and SR-specific protein kinases (SRPKs). As one of the CLKs family member, CLK1 bears a catalytic domain at residues 148–484 aa, and structurally exhibits a typical protein kinase fold [4]. Through phosphorylation of its C-termini, human CLK1 controls alternative RNA splicing by regulating the cellular distribution and splicing activity of the SR family splicing factors. Serine/arginine-rich splicing factor 5 (SRSF5; also called SRp40, HRS) is a member of serine/arginine-rich SR family [26], and is critically involved in pre-mRNA alternative splicing and translation [27]. SRSF5 has been reported to be abnormally expressed in a variety of cancers [28–30]. However, the functions of SRSF5 in pancreatic cancer have not been intensively elucidated.

In the present study, we found that CLK1 expression was upregulated in human PC cells at both mRNA and protein levels. In addition, CLK1 overexpression promoted the proliferation, migration, and invasion of PC cells via activating the phosphorylation of SRSF5 on serine 250. Moreover, the CLK1-SRSF5 axis impacted the alternative splicing pattern of METTL14, which further regulated m6A modification, cell migration and invasion in PC cells. Meanwhile, the CLK1-SRSF5 axis also enhanced PC cell proliferation via AS of Cyclin L2. Thus, the CLK1-SRSF5 axis is potentially a prognostic biomarker and a therapeutic target for treating PC patients.

## Methods

### Patients and specimens

Clinical tissue samples from 344 patients were obtained from Fujian Provincial Hospital (Fuzhou, China) and Ruijin Hospital (Shanghai, China). The internal cohort consisting of 186 paired pancreatic tumor tissues and adjacent non-tumor tissues was from Fujian Provincial Hospital, while the external validation cohort comprised 158 pancreatic cancer tumor tissues and adjacent non-tumor tissues from Ruijin Hospital.

All patients were subjected to distal pancreatectomy or pancreaticoduodenectomy between September, 2010 and September, 2015. For each patient, the paraffin-embedded tissue blocks were collected for the generation of tissue microarrays (TMAs). Detailed clinicopathological data of all the patients, such as sex, age, tumor size, tumor stage, microvascular invasion, and lymph node metastasis, were obtained from hospital records. Patients were followed up until March, 2020. All experiments involving human samples and clinical data were approved by the Accreditation Committee of Fujian Provincial Hospital.

### RNA sequencing and public datasets

Fifteen pairs of paired pancreatic tumor tissues and adjacent non-tumor tissues were selected for RNA-sequencing analysis at Amoydx Bio-tech Inc. (Xiamen, China). Genome mapping on the reads for each sample was applied with a transcriptome built from GRCh37/hg19 by using STAR 2.7 and quantified transcript abundances in transcripts per million (TPM) by using RSD v1.3.3. Standard selection criteria to identify differentially expressed RNAs included a *P* < 0.05 and a fold change > 2. The correlation of expression profiles between tumor tissues and adjacent non-tumor tissues was determined by unsupervised hierarchical clustering analysis. The public datasets used in this study included three GEO datasets (http://www.ncbi.nlm.nih.gov/geo/, GSE46234, GSE46385, and GSE71989).

### Immunohistochemistry

Immunohistochemistry staining of TMA sections was performed using antibodies targeting CLK1 (Cat.No.sc-515897, Santa Cruz, USA) and SRSF5 (Cat. No.ab67175, ab67175, Abcam, USA), as previously described [27, 28]. The immuneostained sections were independently scored by two experienced pathologists in a blinded manner. The staining intensities of CLK1 and SRSF5 were assigned as follows: 0, negative; 1, weak; 2, medium; and 3, strong. The positive rate of tumor cells was scored as follows: 1, 0–25%; 2, 26–50%; 3, 51–75%; and 4, 76–100%. The immune response score (IRS) was calculated by multiplying the staining intensity and positive rate scores, and the staining patterns were divided into two categories: Low (IRS: 0–6) and High (IRS: 7–12).

### Establishment of cell lines

Human pancreatic cancer cell lines PANC-1, BxPC-3, PaTu8988T were purchased from American Type Culture Collection (ATCC, Manassas, VA, USA). BxPC-3 cells were maintained in RPMI 1640 medium supplemented with 2.5 g/L glucose, 1 mM sodium pyruvate, and 10% fetal bovine serum. PANC-1 and PaTu8988T cells were cultured in Dulbecco’s modified Eagle’s medium (DMEM) supplemented with 10% FBS and 2 mM L-glutamine. Cells were cultured in a 37 °C, 5% CO_2_ incubator with routine mycoplasma check once every month.

Plasmids for lentivirus packaging were purchased from Shanghai Genechem Inc (Shanghai, China). Plasmids were transfected in HEK-293 T cells by using Lipofectamine 3.0 (Invitrogen) following the manufacturer's instructions. The lentivirus-containing supernatant was collected 48 and 72 h after transfection, and cells were transfected with lentivirus. The pancreatic cancer cell lines with stable gene expression were selected in culture medium supplemented with puromycin (1 μg/ml; Sigma-Aldrich, St. Louis, MO, USA).

### Colony formation assay

Cells were seeded into 12-well plates at an initial density of 700 cells/well and then were cultured for 2 weeks. The colonies were fixed in 4% paraformaldehyde and stained with 0.1% crystal violet (Sigma, St. Louis, MO, USA). The visible colonies were counted using a light microscope. Numbers of colonies in triplicate wells were measured for each treatment group.

### Label-free quantitative phosphorylation proteomics

Label-free quantitative phosphorylation proteomics was performed in Genechem Biotechnology Co., Ltd. (Shanghai, China). Stable scrambled CLK1 and vector Panc-1 cells were constructed. An appropriate amount of SDT lysate was added in the samples. Ultrasound was performed, and samples was boiled in water for 15 min. After centrifugation at 14 000 g for 15 min, the supernatant was taken out of the samples. Protein in the supernatant was quantified by the BCA method. The solution of 600 μg protein was taken out for each sample, and DTT was added to the final concentration of 100 mM. Samples were boiled in water for 5 min, and cooled down to room temperature. 200μL UA Buffer was added into the sample and mixed well, then the samples were transferred into a 30kD ultrafiltration tube. After centrifugation at 12,500*g* for 15 min, the filtrate was discarded, and this step was repeated once.100μL IAA buffer (100 mM IAA in UA) was added in the tube, followed by shaking at 600 rpm for 1 min and subsequently by dark reaction at room temperature for 30 min. After centrifugation at 12,500 g for 15 min, 100 μL UA Buffer was added in and the tubes were centrifuged at 12,500*g* for 15 min. This procedure was repeated twice. 100 μL 40 mM NH4HCO3 solution was added in, and samples were centrifuged at 12 500 g for 15 min. This procedure was repeated twice. 60μL Trypsin buffer (6 μg Trypsin in 40 μL 40 mM NH4HCO3 solution) was added in, and the samples were shaken at 600 rpm for 1 min. Samples were placed at 37 °C for 16–18 h. A new collection tube was used and samples were centrifuged at 12 500 g for 15 min. Then 40 μL 40 mM NH4HCO3 solution was added in, and samples were centrifuged at 12,500*g* for 15 min. The filtrate was collected. The peptides were desalted by a C18 Cartridge, and the peptides were lyophilized and enriched by phosphorylated peptides. Each sample was separated using the Easy NLC system with a nanoscale flow rate. Buffer solution A is 0.1% formic acid aqueous solution, and buffer solution B is 0.1% formic acid acetonitrile aqueous solution (acetonitrile is 80%). The chromatographic column was balanced with 100% liquid A, and the samples were loaded into the analytical column (Thermo Fisher Scientific, Acclaim Pepmap RSLC 50 μm × 15 cm, Nano Viper, P/N164943) at a flow rate of 300 nL/min by automatic sampler. After chromatographic separation, the samples were analyzed by mass spectrometry with Q ExActive Plus. The duration of analysis was 60/120/180 min (according to the specific experimental scheme), the detection method was positive ion. The scanning range of mother ion was 350–1800 m/z, and the resolution of primary mass spectrometry was 70,000. The AGC target was 3^6, and the primary Maximum IT was 50 ms. The mass charge ratio of peptides and peptide fragments were collected according to the following methods: MS2 scan was collected after each full scan; MS2 Activation Type was HCD; Isolation window was 2 m/z; secondary mass spectrometry resolution was 17,500; microscan was 1; secondary Maximum IT was 120 ms; and Normalized Collision Energy was 30 eV.

### Transcriptome sequencing

Total RNA was extracted from the indicated PANC-1 cells with TRIzol Reagent (Invitrogen, Carlsbad, CA, USA). Transcriptome sequencing was performed at Genechem Biotechnology Co., Ltd. (Shanghai, China) following the standard experimental procedures as below.

#### RNA quantification and qualification

RNA degradation and contamination were monitored on 1% agarose gels. RNA purity was checked using the NanoPhotometer ® spectrophotometer (IMPLEN, CA, USA). RNA integrity was assessed using the RNA Nano 6000 Assay Kit of the Bioanalyzer 2100 system (Agilent Technologies, CA, USA).

#### Library preparation for Transcriptome sequencing

A total amount of 1 µg RNA per sample was used as input material for the RNA sample preparations. Sequencing libraries were generated using NEBNext® UltraTM RNA Library Prep Kit for Illumina® (NEB, USA) following manufacturer’s recommendations and index codes were added to attribute sequences to each sample. Briefly, mRNA was purified from total RNA using poly-T oligo-attached magnetic beads. Fragmentation was carried out using divalent cations under elevated temperature in NEBNext First Strand Synthesis Reaction Buffer (5X). First strand cDNA was synthesized using random hexamer primer and M-MuLV Reverse Transcriptase (RNase H-). Second strand cDNA synthesis was subsequently performed using DNA Polymerase I and RNase H. Remaining overhangs were converted into blunt ends via exonuclease/polymerase activities. After adenylation of 3′ ends of DNA fragments, NEBNext Adaptor with hairpin loop structure was ligated to prepare for hybridization. In order to select cDNA fragments of preferentially 250 ~ 300 bp in length, the library fragments were purified with AMPure XP system (Beckman Coulter, Beverly, USA). Then 3 µl USER Enzyme (NEB, USA) was used with size-selected, adaptor-ligated cDNA at 37 °C for 15 min followed by 5 min at 95 °C before PCR. Then PCR was performed with Phusion High-Fidelity DNA polymerase, Universal PCR primers and Index (X) Primer. At last, PCR products were purified (AMPure XP system) and library quality was assessed on the Agilent Bioanalyzer 2100 system.

#### Clustering and sequencing

The clustering of the index-coded samples was performed on a cBot Cluster Generation System using TruSeq PE Cluster Kit v3-cBot-HS (Illumia) according to the manufacturer’s instructions. After cluster generation, the library preparations were sequenced on an Illumina Novaseq platform and 150 bp paired-end reads were generated.

#### Transcriptome sequencing-data analysis

Raw data (raw reads) of fastq format were firstly processed through in-house perl scripts. In this step, clean data (clean reads) were obtained by removing reads containing adapter, reads containing ploy-N and low-quality reads from raw data. At the same time, Q20, Q30 and GC content of the clean data were calculated. All the downstream analyses were based on the clean data with high quality. Reference genome and gene model annotation files were downloaded from genome website directly. Index of the reference genome was built using Hisat2 v2.0.5 and paired-end clean reads were aligned to the reference genome using Hisat2 v2.0.5. We selected Hisat2 as the mapping tool for that Hisat2 can generate a database of splice junctions based on the gene model annotation file and thus a better mapping result than other non-splice mapping tools. Feature Counts v1.5.0-p3 was used to count the reads numbers mapped to each gene. And then FPKM of each gene was calculated based on the length of the gene and reads count mapped to this gene. FPKM, expected number of Fragments Per Kilobase of transcript sequence per Millions base pairs sequenced, considers the effect of sequencing depth and gene length for the reads count at the same time, and is currently the most used method for estimating gene expression levels. Gene Ontology (GO) enrichment analysis of differentially expressed genes was implemented by the cluster Profiler R package, in which gene length bias was corrected. GO terms with corrected *P* value less than 0.05 were considered significantly enriched by differential expressed genes. KEGG is a database resource for understanding high-level functions and utilities of the biological system, such as the cell, the organism, and the ecosystem, from molecular-level information, especially large-scale molecular datasets generated by genome sequencing and other high-throughput experimental technologies (http://www.genome.jp/kegg/). We used cluster Profiler R package to test the statistical enrichment of differential expression genes in KEGG pathways.

#### AS analysis

rMATS(3.2.5) software was used to analysis the AS event.

### RNA immunoprecipitation (RIP)

Immunoprecipitations targeting CCNL2 and METTL14 pre-mRNA were performed using aMagna RIP™ (Catalog No. 17-700, MilliporeSigma, USA) according to the manufacturer’s protocol. The immunoprecipitated RNA extracts were reverse-transcribed using the TRIzol Reagent (Invitrogen, Carlsbad, CA, USA), and the quantification of target RNA was performed by real-time quantitative PCR (qPCR).

### RNA pulldown assay

The biotinylated METTL14 and CCNL2 RNA baits were purchased from the Genepharma Corporation (Shanghai, China). RNA pulldown assays were performed using a MagCapture™ RNA Pull Down kit (Millipore Corporation, USA) per the manufacturer’s instructions. Protein quantification in the immunoprecipitated protein extracts was conducted by Western blot assay.

#### Cross-linking immunoprecipitation q-PCR(CLIP-qPCR)

Crosslinking-immunoprecipitation was performed in Wuhan IGENEBOOK Biotechnology Co., Ltd (http://www.igenebook.com). Briefly, one hundred million cells expressing Flag-SRSF5 were irradiated with 254 nm UV-C light at 400 mJ/cm^2^ on ice to cross-link proteins to nucleic acids. Protein-RNA complexes were obtained after cell lysis and immunoprecipitated with anti-Flag antibody (F1804, Sigma) or mock immune-precipitated with IgG (2729, CST). After high-salt stringent washing, protein-RNA complexes run on 4–12% NuPAGE (Invitrogen) and protein-RNA complexes in the range of 10–70 kDa were recovered and digested with proteinase K. After transcription reversing, cDNA was obtained amplified by PCR using their specific primers. PCR reactions were set up and run using the ChamQ SYBR Color qPCR Master Mix. The enrichment values were normalized to the input sample and calculated using the 2^(−ΔΔCt) method.

#### Immunoprecipitation and Western blot assay

HEK-293T cells were transfected with plasmids carrying Flag tagged CLK1 (Flag-CLK1) cDNA and HA tagged SRSF5 (HA-SRSF5) cDNA. At 24 h after transfection, cells were harvested to assess the protein–protein interaction between CLK1 and SRSF5. For immunoprecipitation and Western blot assay, the nuclear and total cellular protein fractions were extracted with RIPA Lysis Buffer (Beyotime, China), as previously described [28]. The primary antibodies used in this study included anti-Flag (Cat. No. F7425, Sigma-Aldrich, USA), anti-HA (Cat.No.MMS-101P, Biolegend, USA), anti-CLK1 (Cat.No.sc-515897, Santa Cruz, USA), anti-SRSF5 (Cat. No. ab67175, ab67175, Abcam, USA), E-cadherin (Cat. No. ab231303, Abcam, UK), anti-P21 (Cat. No.10355–1-AP, Proteintech, USA), anti-CDK4 (Cat. No. ab108357, Abcam), and anti-N-cadherin (Cat. No. ab207608, Abcam) antibodies. The anti-phosphorylation –SRSFs was constructed in GenScript Inc (Nanjing, China).

#### Animal experiments

Male BALB/c nude mice (nu/nu, 5-week old) were purchased from SLAC Laboratory Animal Co. Ltd. (Shanghai, China), and housed at the specific pathogen-free (SPF) facility at the Animal Center of Fujian Medical University at room temperature (22 ± 1 °C) with a 12/12 h light/dark cycle and access to food and water ad libitum. A total of 4 × 10^6^ stably transfected cells with genetically altered expression of CLK1 or SRSF5 were subcutaneously injected into the right axillary fossa of the nude mice (eight mice per group). Tumor length (*L*) and width (*W*) were measured every 4 days, and tumor volume was calculated as 0.5 × *L* × *W*^2^. The mice were sacrificed at 4–5 weeks after tumor inoculation, and the tumors were weighed, photographed, and harvested for further characterizations.

Luciferase-expressing cells were used to establish the lung metastasis model. Briefly, PANC-1-luc2/Neo or BxPC-3-luc2/Neo cell line (with stable expression of luciferase, from Promega Corporation, USA) was infected with lentivirus packaged from empty lentiviral vector, or control shRNA plasmids. These cells were then injected into the tail veins of nude mice at a dose of 5 × 10^6 cells/mouse. At 5 weeks after tumor cell injection, bioluminescence imaging was conducted to track lung metastasis. For Survival analysis, nude mice were followed up for 10 weeks after inoculation of tumor cells with altered expression levels of CLK1 and SRSF5. All the animal experiments were approved by Animal Welfare Committee of Fujian Medical University (Fuzhou, China).

#### Statistical analysis

Statistical analyses were performed using the SPSS software (version 17.0; IBM, Armonk, NY, USA). Differences between the indicated groups were compared using the t-tests and one-way analysis of variance (ANOVA) followed by Fisher's least significant difference (LSD) test. The cumulative overall survival (OS) and disease-free survival (DFS) rates were calculated using the Kaplan–Meier method, and differences between curves were evaluated using the log-rank test. A *P* value < 0.05 was considered to indicate a statistically significant result.

## Results

### Elevated expression of CLK1 in pancreatic cancer tissues correlated with worse prognosis of PDAC patients

**Fig. 1:**
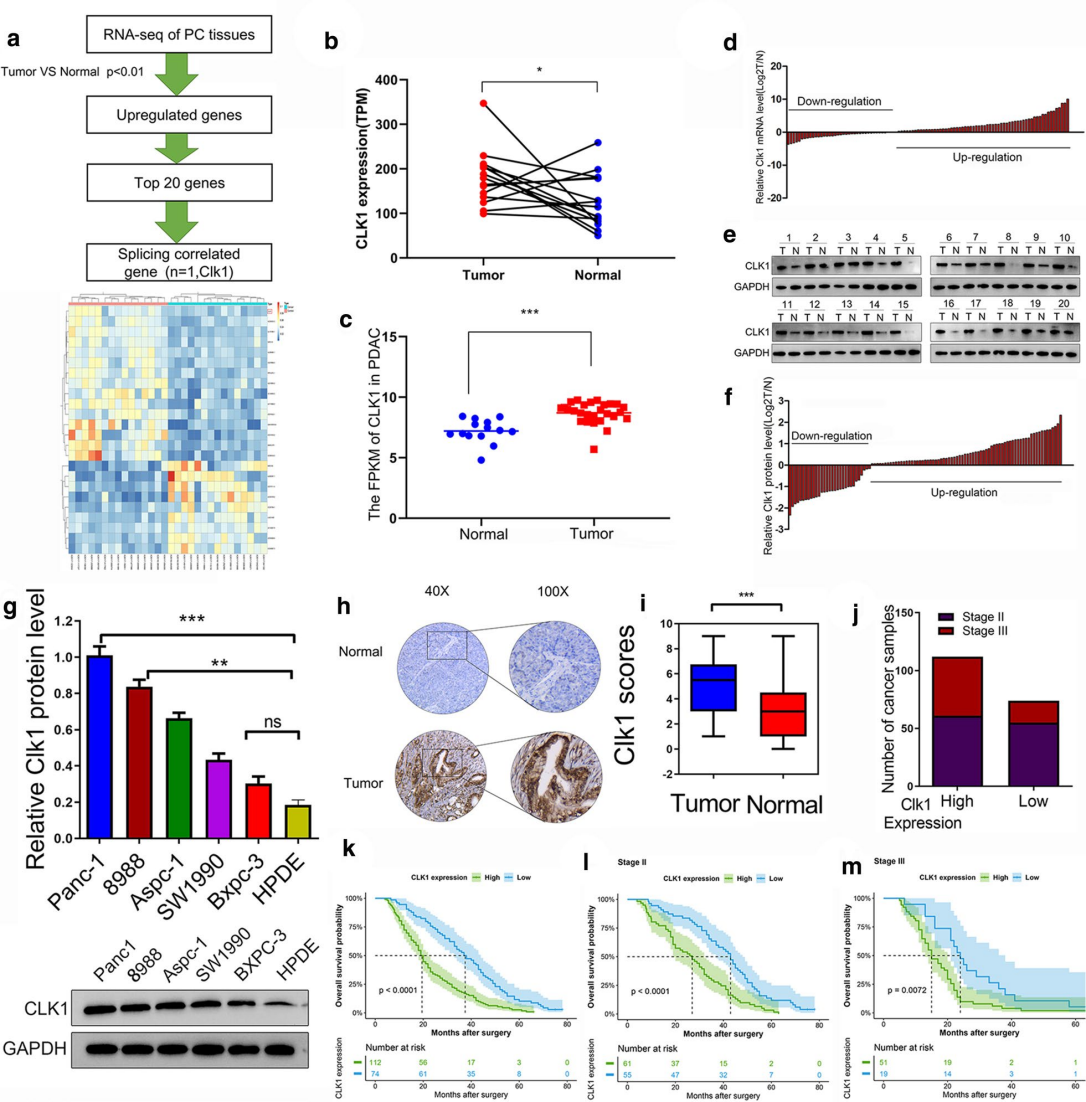
The expression and prognostic value of CLK1 in Human pancreatic cancer. **a** Flowchart showing the screening process of candidate genes by RNA-seq of normal tissues and PC tissues and alternative splicing analyses. **b**, **c** The TPM (transcripts per million (**b**); and FPKM (fragments per kilobase million (**c**)) of CLK1 in pancreatic tumor and adjacent normal tissues from GEO database were analyzed. n = 15 for normal tissues, n = 26 for tumor tissues. **d** The mRNA levels of CLK1 in 102 pairs of PDAC tumor and adjacent normal tissues were measured by qPCR. **e**, **f** Representative images of CLK1 protein levels in PDAC tumor and adjacent normal tissues by western blot assays (**e**). The changes of CLK1 protein expression in 103 pairs normal and tumor tissues were summarized (**f**). **g** The protein levels of CLK1 in normal human pancreatic duct epithelial (HPDE) cells and selected human pancreatic cancer cell lines were quantitated by western blot assays. Representative images are shown, and relative expression levels were summarized. **h**, **i** The expression of CLK1 in 186 paraffin-embedded specimens from the internal cohort was determined by TMA-based IHC staining. Representative IHC images are shown (**h**), and the relative CLK1 staining intensity was scored (**i**). Scale bar, 200 μm. **j** The number of PDAC tissues with high or low expression levels of CLK1 from patients with Stage III and Stage II pancreatic cancer was summarized. **k**–**m** Kaplan–Meier analyses of the correlations between CLK1 expression and overall survival of all PDAC patients (**k**), or Stage II patients (**l**), or Stage III patients (**m**) in the internal cohort. Data are shown as mean ± SD from three independent experiments. *P < 0.05; **P < 0.01; ***P < 0.001, between the indicated groups

High-throughput sequencing was performed in specimens consisting of 15 pairs of paired PDAC and pancreatic normal tissues. CLK1 was identified as the only one splicing correlated over-expression gene (Fig. 1a), and was selected for subsequent experiments. CLK1 displayed a higher level of transcript in the majority of PDAC tissues than the paired normal tissues (Fig. 1b). Results from the GEO and TCGA database also confirmed that expression of CLK1 was more in PDAC tissues than that in normal pancreatic tissues (Fig. 1c and Additional file 1: Figure S1A). In addition, further quantitation of CLK1 mRNA (Fig. 1d) and protein (Fig. 1e, f) revealed that pancreatic cancer tissues had higher levels of CLK1 expression than the adjacent non-tumor tissues. Moreover, compared to the normal human pancreatic duct epithelial (HPDE) cells, human pancreatic cancer cell lines including PANC-1, 8988, Aspc-1, SW1990, BxPC-3 had more CLK1 protein expression. While PANC-1 cells exhibited the highest level of CLK1, BxCP-3 cells showed the lowest level of CLK1 among these cancer cell lines (Fig. 1g).

To further evaluate the relationship between expression of CLK1 and clinicopathological features in pancreatic cancer, we determined CLK1 protein expression in paraffin-embedded pancreatic cancer specimens from 186 patients using IHC (Fig. 1h). CLK1 expression was scored as high in 112 (60.2%) and low in 74 (39.8%) samples, and tumor tissues had significantly higher CLK1 expression than normal tissues (Fig. 1i). Notably, the PDAC patients at stage III had significantly more cases of tumor tissues with CLK1-high expression, while the difference in CLK1 expression levels (high vs low) was not obvious in the patients at stage II (Fig. 1j). Univariate analysis showed that the increased CLK1 expression indicated by IHC was associated with tumor size, lymphatic metastasis, and TNM stage (Table 1). Moreover, the overall survival rate (Fig. 1k) for patients with a high CLK1 expression in tumor tissues was significantly lower than that for patients with a low CLK1 expression. In addition, survival analysis in patients at PDAC stage II (Fig. 1l) and stage III (Fig. 1m) also confirmed that a higher CLK1 expression in tumors predicted worse prognosis of PDAC patients. Similar results were obtained in the external validation of another cohort of patients from Ruijin Hospital (Additional file 1: Figure S1B-I). Taken together, these results suggest that elevated CLK1 expression in pancreatic cancer correlated with worse prognosis of PDAC patients.

**Table 1:** Correlation between CLK1 expression and clinicopathological characteristics of PDAC patients

	Internal cohort (n = 186)					External cohort (n = 158)				
		High CLK1 n = 112	Low CLK1 n = 74	*χ*^2^	*p*		High CLK1 n = 96	Low CLK1 n = 62	*χ*^2^	*p*
Age										
< 60	90	50	40	1.58	0.29	94	56	38	0.14	0.42
> 60	96	62	34			64	40	24		
Gender										
Male	99	61	38	0.17	0.68	85	52	33	0.013	0.91
Female	87	51	36			73	44	29		
BMI										
≥ 28	53	33	20	0.13	0.72	62	36	26	0.31	0.58
< 28	133	79	54			96	60	36		
Smoke										
Yes	64	40	24	0.21	0.65	86	56	30	1.50	0.22
No	122	72	50			72	40	32		
Differentiation										
Low	91	49	42	3.02	0.08	77	48	29	0.16	0.69
High/middle	95	63	32			81	48	33		
Lymphatic metastasis										
Yes	126	85	45	4.817	0.028	88	59	26	5.776	0.016
No	60	27	29			70	37	36		
Tumor size										
> 4 cm	71	46	19	4.646	0.031	50	40	11	9.864	0.002
≤ 4 cm	115	66	55			108	56	51		
Microvascular invasion										
Yes	60	42	18	3.54	0.06	64	37	27	0.39	0.53
No	126	70	56			94	59	35		
ki-67										
≥ 20	74	48	26	1.11	0.29	69	43	26	0.13	0.72
< 20	112	64	48			89	53	36		
TNM stage										
II	116	61	55	7.49	0.006	96	53	45	4.83	0.028
III	70	51	19			62	43	17		

^*^P < 0.05 was considered significant

### CLK1 promoted the growth of human pancreatic cancer cells in vitro and in vivo

**Fig. 2:**
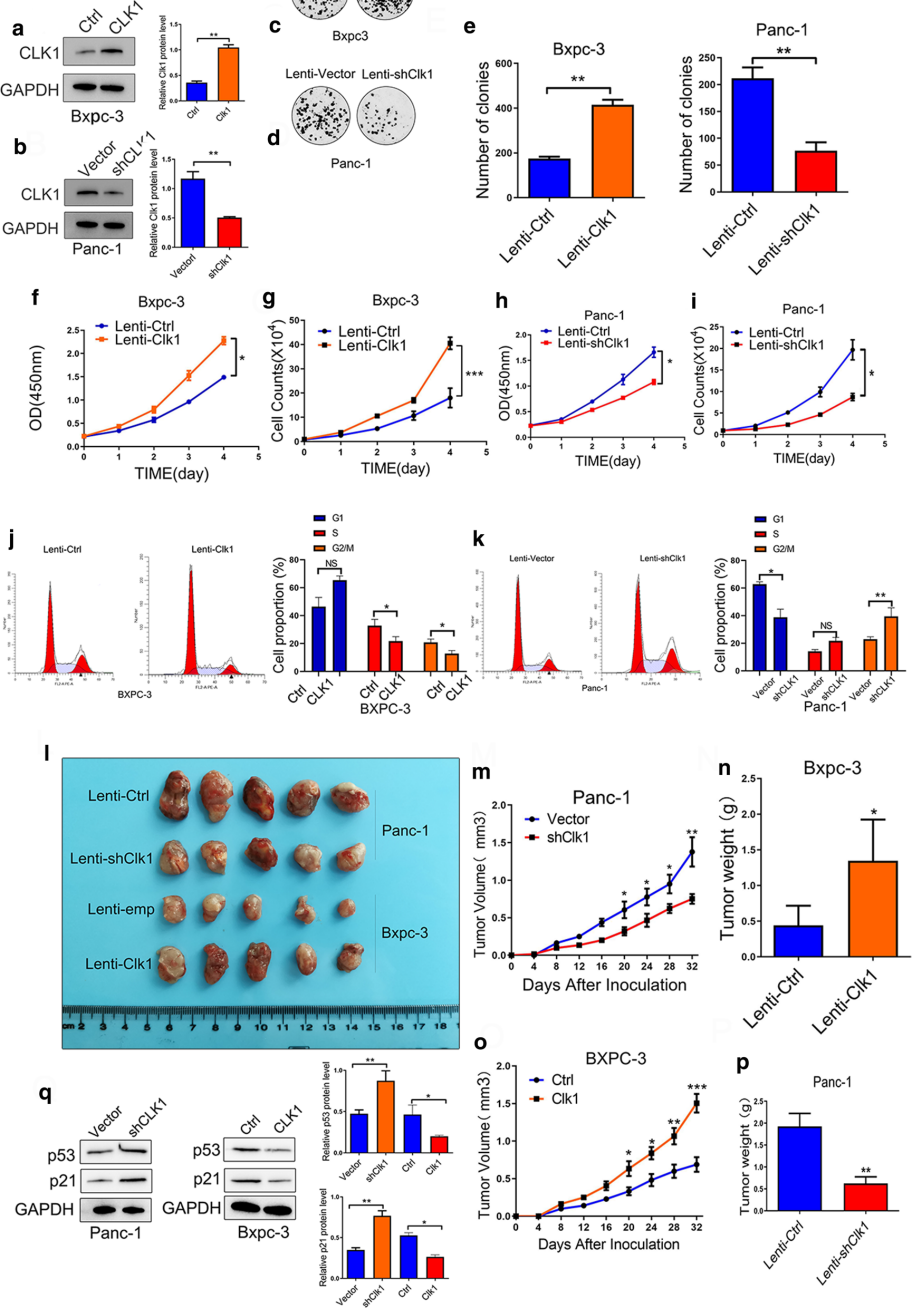
CLK1 enhanced the proliferation ability of human pancreatic cancer cells in vitro and in vivo. **a**, **b** BxPC3 cells with stable CLK1 overexpression (**a**) and PANC-1 cells with CLK1 knockdown (**b**) were generated. The changes in CLK1 expression were confirmed using western blot assays. **c–i** The proliferative ability of stably transfected PANC-1 or BxPC3 cells was investigated via colony formation assays (**c**–**e**) and CCK-8 assays (**f**–**i**). Representative colony formation images are shown (**c**, **d**), and the numbers of colonies were summarized (**e**). **j**, **k** Flow cytometry analysis of the cell cycle progression of stably transfected PANC-1 (**k**) or BxPC3 (**j**) cells was performed. Representative images and quantification of the results are presented. **l**–**n** Overexpression of CLK1 promoted the growth of BxPC-3 cells in a subcutaneous xenograft mouse model (**l**–**n**). The size of the tumors was measured at the indicated time points (**m**, ***P < 0.001). Tumors were extracted and weighed after mice were sacrificed (**n**, ***P < 0.001). CLK1 knockdown inhibited the growth of PANC-1 cells in a subcutaneous xenograft mouse model (**l**, **o**, **p**). The size of the tumors was measured at the indicated time points (**o**, ***P < 0.001). Tumors were extracted and weighed after mice were sacrificed (**p**, ***P < 0.001). **q** The protein levels of p21 and p53 were quantitated by western blot assays, and representative images are shown. Data are shown as mean ± SD from three independent experiments. *P < 0.05; **P < 0.01; ***P < 0.001, between the indicated groups

To examine the effect of altered CLK1 expressions on pancreatic cancer growth, PANC-1 cells with a high expression level of CLK1 and BxPC-3 cells with a lower expression level of CLK1 were selected. First, we generated BxPC-3 cells with stably overexpressed CLK1 (Fig. 2a) and PANC-1 cells with stably knocked-down CLK1 (Fig. 2b). Moreover, 8988 cells with staby overexpressed or knocked-down CLK1 were generated (Additional file 2: Figure S2A, F). The colony formation experiments showed that increased CLK1 expression in BxPC-3 cells (Fig. 2c, E) and 8988 cells (Additional file 2: Figure S2B) significantly promoted cell proliferation, while reduced CLK1 expression in PANC-1 cells (Fig. 2d, e) and 8988 cells (Additional file 2: Figure S2G) significantly suppressed cell proliferation. Similar patterns were observed in CCK-8 (Fig. 2f, h, Additional file 2: Figure S2C, S2H) and cell counts (Fig. 2g, i, Additional file 2: Figure S2D, S2I) assays, suggesting the proliferative role of CLK1 in human pancreatic cancer progression in vitro. In addition, our flow cytometry experiments demonstrated that the main effect of CLK1 on cell proliferation was associated with perturbation of cell cycle, as ectopic CLK-1 expression decreased the percent of cells at G2/M phase (Fig. 2j), while CLK-1 knockdown induced the opposite effects on cell cycle phases (Fig. 2k).

Next, we evaluated the effect of manipulated CLK1 expressions on pancreatic cancer cell growth in vivo (Fig. 2l). Consistent with the in vitro results, xenograft tumors with a high CLK1 expression level grew faster than the control xenografts (Fig. 2m, n), whereas xenografts with downregulated CLK1 expression grew more slowly than the control xenografts (Fig. 2o, p). Moreover, we also determined the levels of key molecules involved in regulating cell cycle progression, and found that the expressions of p53 and p21 were negatively correlated with the expression of CLK1 in these cells (Fig. 2q), and we also found that the expressions of p53 and p21 were inhibited when CLK1 was overexpressed in xenografted tumors (Additional file 2: Figure S2K-M). Collectively, our results indicated that CLK1 acted as an oncogenic factor through promoting the proliferation of pancreatic cancer cells both in vitro and in vivo.

### CLK1 promoted the migration and invasion ability of human pancreatic cells in vitro and in vivo

**Fig. 3:**
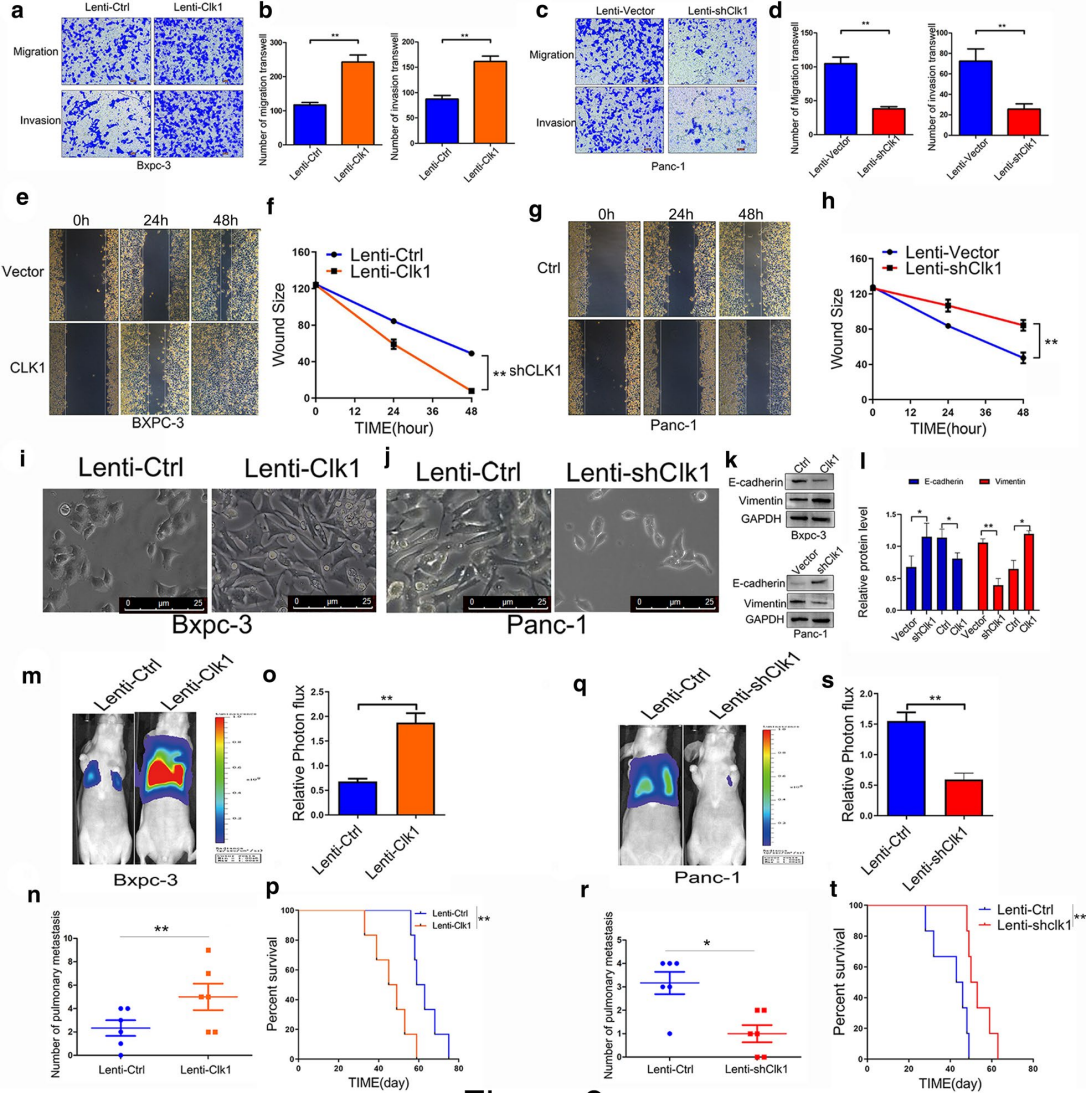
CLK1 promoted the migration and invasion ability of pancreatic cells in vitro and in vivo. **a**–**d** Transwell assays with stably transfected BxPC-3 (**a**, **b**) and PANC-1 (**c**, **d**) cells were performed. Representative images and quantification of the results are presented. **e**–**h** Wound-healing assays with stably transfected BxPC-3 (**e**, **f**) and PANC-1 (**g**, **h**) cells were performed. Representative images and quantification of wound closure are presented. **i**–**l** The epithelial–mesenchymal morphology of BxPC-3 (**i**) and PANC-1 (**j**) cells, scale bars for the images are 25 μm. And the protein expressions of E-cadherin, and Vimentin were analyzed, the quantification are presented on the right (**k**, **l**). **m**–**t** Control BxPC-3 cells or CLK-1-overexpressing BxPC-3 cells (**m**–**p**), or control PANC-1 cells, or CLK-1-knocked down PANC-1 cells (**q**–**t**) were injected into nude mice via tail vein, and tumor metastasis to lung was evaluated after 6 weeks. Representative bioluminescence images of mice are presented (**m**, **o**, **q**, **s**), and the number of pulmonary metastasis (**n**, **r**) was summarized. Mice survival analysis was further analyzed (**p**, **t**). Data are shown as mean ± SD from three independent experiments. *P < 0.05; **P < 0.01; ***P < 0.001, between the indicated groups

To investigate the role of CLK1 in PDAC metastasis, we used the PC cell lines with stable overexpression or knock-down CLK1 to conduct migration and invasion assays. Results from transwell assays experiments showed that elevated CLK1 expression in BxPC-3 cells (Fig. 3a, b) and 8988 cells (Additional file 3: Figure S3A-3C) significantly promoted cell migration and invasion, while reduced CLK1 expression in PANC-1 cells (Fig. 3c, d) and 8988 cells (Additional file 3: Figure S3E-3G) showed the opposite effects. Similar results were observed in wound healing assays, which demonstrated that the expression of CLK1 was clearly positively related to the migration and invasion ability of BxPC-3 cells (Fig. 3e, f), PANC-1 cells (Fig. 3g, h), and 8988 cells (Additional file 3: Figure S3D,3H) in vitro. Moreover, we also found that CLK1 overexpression caused the transition of BxPC-3 cells to spindle-shaped mesenchymal cells (Fig. 3i), while the adhesion between the tumor cells was enhanced. Quantification of EMT makers showed that CLK1 overexpression was associated with reduced expression of E-cadherin and increased expressions of Vimentin in Bxpc-3 cells (Fig. 3k). As expected, CLK-1 knockdown in PANC-1 cells resulted in the opposite effects on cell morphology and EMT (Fig. 3j, l).

In order to explore the effect of altered CLK1 expressions on the processes of invasion and metastasis of PC cells in vivo, BxPC-3 cells with CLK1 overexpression or PANC-1 cells with CLK1 knock-down and the corresponding control cells were injected into nude mice to track their metastasis. At 4 weeks after tumor cells inoculation, mice injected with CLK1 overexpressing cells exhibited enhanced lung metastasis, as evidenced by bioluminescence imaging (Fig. 3m, o), while impaired lung metastasis was observed when CLK1 knock-down cells were inoculated (Fig. 3q, s). In addition, histological analysis of the dissected lungs confirmed that the CLK1-overexpression group had more metastatic nodules than the control group (Fig. 3n), while CLK1-knockdown group exhibited an opposite effect (Fig. 3r).Moreover, we also found that mice with CLK1 overexpression in xenografted BxPC-3 tumors had reduced overall survival (Fig. 3p), while mice with CLK1 depletion in xenografted PANC-1 tumors displayed prolonged survival in our mouse model (Fig. 3t), in comparison to the control groups. Therefore, our results indicated that CLK1 expression promoted the metastatic ability of human pancreatic cancer cells in vitro and in vivo.

### CLK1 was widely involved in phosphorylation regulation of alternative splicing related proteins, especially SRSF5 ^250 serine^

**Fig. 4:**
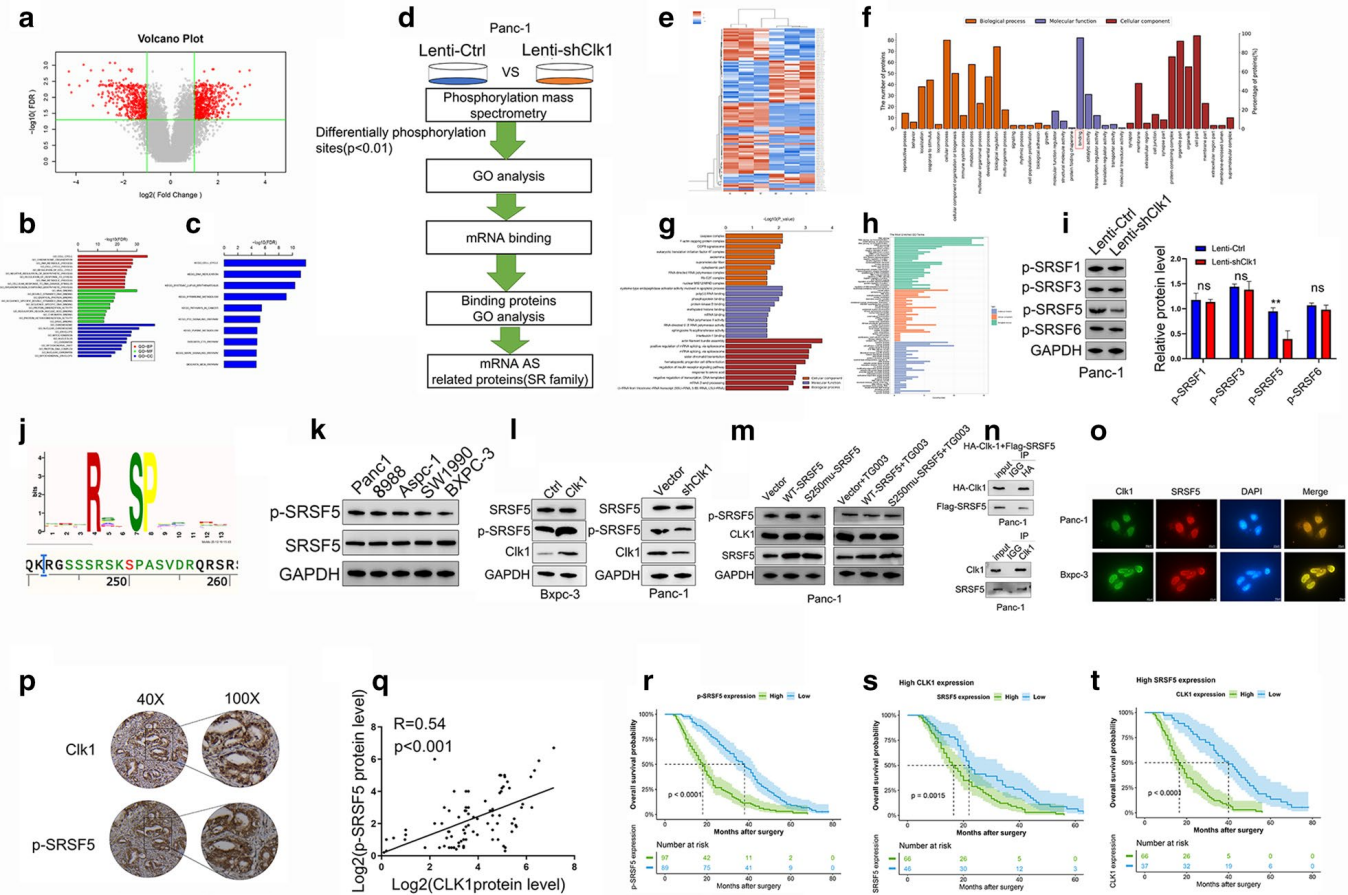
CLK1 phosphorylates SRSF5 on Serine 250 in human pancreatic cancer cells. **a** Volcano plot of differential expression genes between CLK-KD and CLK1-vector groups. **b**, **c** GO and KEGG analysis of the DEGs was performed and results were represented. **d** Flowchart showing the process of evaluating the involvement of CLK1 in regulating alternative splicing related proteins. **e** Phosphorylation mass spectrometry was performed, and clusters plots represents differentially phosphorylation expressed sites (DPS) between CLK1-konckdown and control cells. **f**–**h** GO analysis of the DPS related proteins was performed and results were represented. **i** Changes of phosphorylation in the splicing factors (SRSF1, SRSF3, SRSF5, SRSF6) screened by phosphorylation mass spectrometry were confirmed by western blot assays. **j** Logo pictures represented the 250-Ser of SRSF5. **k** Relationship between CLK1 expression and phosphorylation level of SRSF5 on Ser250 in different cell lines and **l** CLK overexpressed or knockdown cell lines was determined by western blot assays. **m** CLK1 activated the phosphorylation level of SRSF5 on Ser250. **n** Up, the interaction between CLK1 and SRSF5 was analyzed in Panc-1 cell transfected with plasmids encoding HA-CLK1 and Flag-SRSF5 by co-immunoprecipitation and western blot assays. Bottom, Endogenous interaction between CLK1 and SRSF5 was assayed in PANC-1 cell lysates by co-im0munoprecipitation and western blot assays using an anti-CLK1 or anti-SRSF5 antibody, respectively. Ten percent of the input was loaded, and normal IgG was used as a control. **o** Immunofluorescence staining of CLK1 (green), SRSF5 (red) and DAPI (blue) in PANC-1 and BxPC3 cells was conducted. Scale bars for the images are 50 μm. **p** The expressions of phosphorylated SRSF5 and CLK1 in tumor tissues of PDAC patients were evaluated by IHC. **q** The correlation between P-SRSF5 and CLK1 levels in tumor tissues was analyzed. **r**–**t** Kaplan–Meier analyses of the correlations between P-SRSF5 (**r**), SRSF5 in the condition of High CLK1 expressions (**s**) CLK1 in the condition of High SRSF5 expressions (**t**) and overall survival of PDAC patients. Data are shown as mean ± SD from three independent experiments. *P < 0.05; **P < 0.01; ***P < 0.001, between the indicated groups

To explore the underlying mechanism of CLK1 in pancreatic cancer progression, we firstly determined the overall impact of differentiated CLK1 expressions on whole gene expression levels (Fig. 4a). Further GO (Fig. 4b) and KEGG (Fig. 4c) analysis confirmed that the genes differentially expressed in regulation of CLK1 were mainly involved in regulation of cell cycle, which was consistent with our previous verification. Since CLK1 has been reported to be a phosphorylation kinase and alternative splicing factor [31, 32], we performed a lable-free phosphorylation mass spectrometry (Fig. 4d) to explore the target phosphorylated protein of CLK1. The differentially expressed phosphorylation sites with up-regulation in 39 genes and down-regulation in 62 genes were identified (Fig. 4e). GO analysis of the represented proteins showed that CLK1 mainly regulates the phosphorylation status of mRNA alternative splicing-related proteins (Fig. 4f, g). Further GO analysis of the GO term binding confirmed that the differentially phosphorylation status proteins were mainly involved in regulation of mRNA splicing (Fig. 4h). Therefore, the SR protein family closely related to splicing attracted our attention. Of all the proteins whose phosphorylation levels were down-regulated, we found that the phosphorylation level of SRSF5 on Ser-250 was significant. We further verified this finding by western blot assay (Fig. 4i), and the results showed that CLK-1 knockdown in PANC-1 cells led to a decreased level of p-SRSF5 on Ser-250 (Fig. 4j), but not other SRSF5 family members including p-SRSF1, p-SRSF3, and p-SRSF6 (Fig. 4i). In the meantime, the phosphorylation level of SRSF5-ser-250 was correlated with the expression level of CLK1 in different PC cell lines (Fig. 4k and Additional file 4: Figure S4I.A), Also, When CLK1 was knocked down in PANC-1 cells, the phosphorylation level of SRSF5-ser-250 decreased, while CLK1 overexpression in BxPC3 cells led to increased phosphorylation of SRSF5 (Fig. 4l and Additional file 4: Figure S4I.B),Moreover, we constructed SRSF5-ser-250 wild-type/mutation plasmids and transfected into Panc1 cells, we found SRSF5-ser-250 wild-type while not mutation could significantly increased the phosphorylation levels of SRSF5-ser-250, and this effect was eliminated when added CLK1 phosphorylation inhibitor TG003 (Fig. 4m and Additional file 4: Figure S4I.C), Moreover, when we transfected these two plasmids into Bxpc-3 cells, There was no significant changes observed in the phosphorylation levels of SRSF5-ser-250 (Additional file 4: Figure S4I.D and E). Furthermore, results from co-immunoprecipitation (Co-IP) experiments demonstrated the protein–protein interaction between exogenously expressed HA-CLK1 and Flag-SRSF5 in PANC-1 and BxPC-3 cells (Fig. 4n and Additional file 5: Figure S4II A-E). The interaction between endogenous CLK1 and SRSF5 was also confirmed using Co-IP assays (Fig. 4n). Moreover, immunofluorescent microcopy experiments demonstrated co-localizations of CLK1 and SRSF5 in the nucleus of PANC-1 and BxPC-3 cells (Fig. 4o), suggesting that CLK1 may regulate SRSF5 phosphorylation via protein interaction.

Additionally, we further verified the correlation between CLK1 expression and phosphorylation of SRSF5 on 250Ser. We found that the level of phosphorylated SRSF5 elevated in tumor tissues with a high expression level of CLK1 by IHC (Fig. 4p). Moreover, a remarkable positive correlation between CLK1 expression and SRSF5 phosphorylation in our clinical database was noted by western blot (Fig. 4q). Furthermore, high expressions of p-SRSF5 (Fig. 4r and Additional file 4: Figure S4I.F) were associated with worse overall survival or disease-free survival of PC patients, in comparison to the patients with low expressions of p-SRSF5 in tumors.

Next, we evaluated the prognostic value of the combined expression levels of CLK1 and SRSF5. The results showed that when CLK1 was at a high expression level, pancreatic cancer patients with high expression levels of SRSF5 have a poorer prognosis than those with low SRSF5 expression levels (Fig. 4s, t, Additional file 4: Figure S4I.G,H). When CLK1 staining was reduced in the nucleus, no significant survival difference was observed between the patients with low or high SRSF5 expression levels (Additional file 4: Figure S4I.I, 4I.J). The similar effect was observed when SRSF5 staining was down expression in the nucleus, it also no significant survival difference was observed between the patients with low or high CLK1 expression level (Additional file 4: Figure S4K, S4L). These results confirmed that the clinical value of the CLK1-SRSF5 axis in the prognosis of pancreatic cancer. Additionally, the survival of the group with high CLK1 and SRSF5 expression was the worst, while the survival of the group with low expression was the best (Additional file 4: Figure S4I M, N).

In summary, the results demonstrated that CLK1 was widely involved in the phosphorylation regulation of alternative splicing related proteins, among which the 250 serine site of SRSF5 may be an potential important target of CLK1.

### CLK1-mediated SRSF5 phosphorylation on Ser250 contributed to the proliferation and metastasis of pancreatic cancer cells

**Fig. 5:**
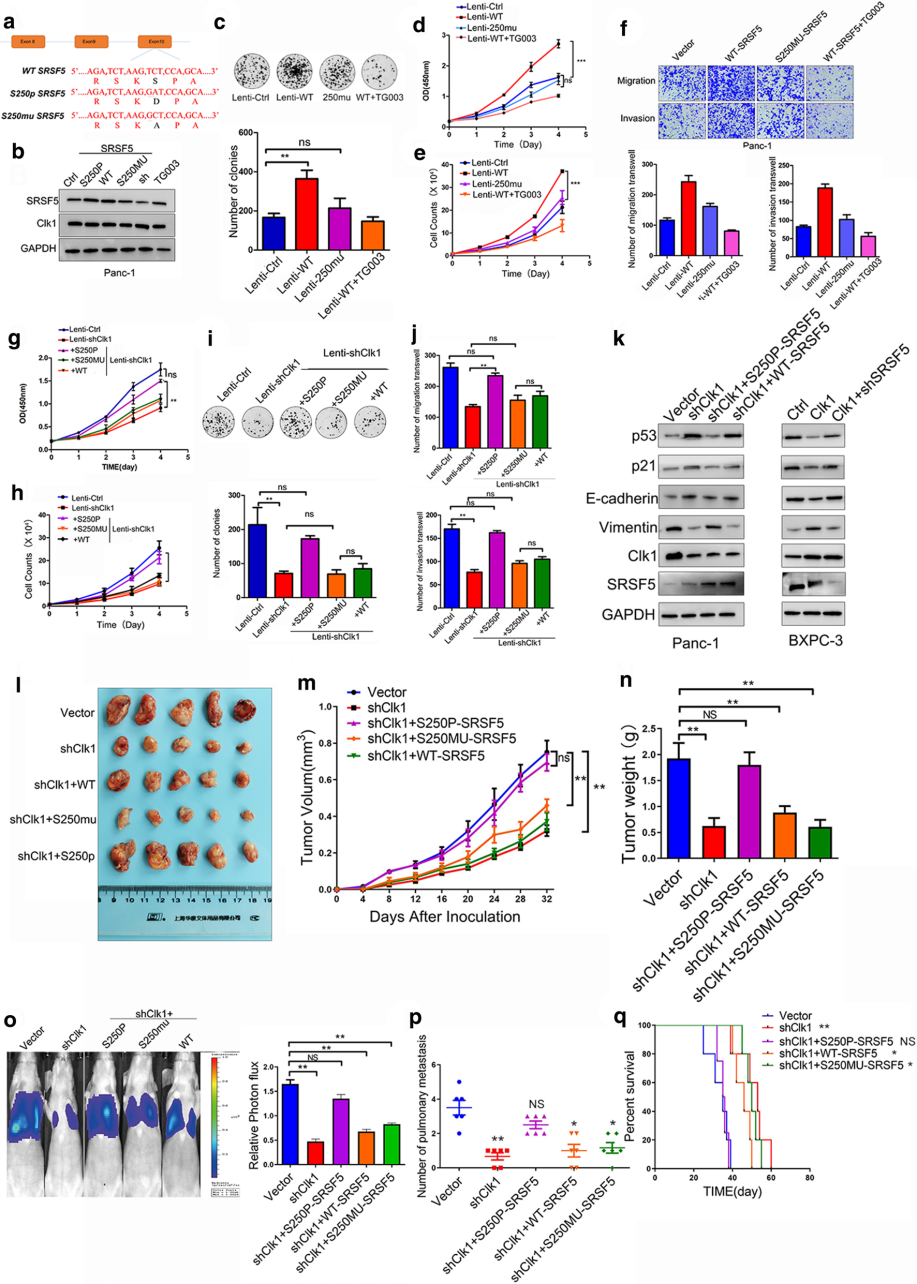
CLK1-induced SRSF5 phosphorylation on Ser250 contributed to the proliferation and metastasis of pancreatic cancer cells. **a** A schematic diagram shows the nuclear and amino acid sequences of wild type, mutated, and phosphorylated SRSF5 at Ser250 site. **b** PANC-1 cells were left untreated (Ctrl), or transduced with plasmids expressing wild type (WT)/mutated (S250MU)/phosphorylated (S250P) SRSF5, or treated with TG003 for 24 h. The protein levels of SRSF5 and CLK1 were determined by western blot assays. **c**–**f** The colony formation (**c**) and proliferation (**d**, **e**) ability, migration and invasion ability (**f**) of the indicated cell lines were evaluated. **g**–**k** PANC-1 cells were infected control lentivirus, or lentivirus expressing CLK1-specific shRNA, or lentivirus expressing CLK1 shRNA together with lentivirus expressing wild type (WT)/mutated (S250MU)/phosphorylated (S250P) SRSF5. The cell proliferation ability (**g**, **h**), colony formation ability (**i**), migration and invasion ability (**j**) of the indicated cells were determined. The protein levels of p21, p53, E-cadherin, and Vimentin in the indicated cells were determined by western blot assays (**k**). **l**–**q** PANC-1 cells as indicated in (**h**) were inoculated into nude mice, and mice were sacrificed. The images of tumors (**l**), bioluminescence images of mice (**o**), and tumor growth curves (**m**), tumor weight (**n**), mice survival (**p**), and number of pulmonary metastasis (**q**) are shown. All data are shown as mean ± SD from three independent experiments. *P < 0.05; **P < 0.01; ***P < 0.001, between the indicated groups

To explore the function of SRSF5 phosphorylation on Ser 250 in regulating the malignant behavior of PDAC cells, we generated lentivirus that can be used to overexpress SRSF5 with the following isoforms: SRSF5 mutation on 250-Ser site (S250MU), simulated phosphorylation on 250-Ser site (S250P), and wild type SRSF5 (S250WT) (Fig. 5a). Lentivirus infection-mediated overexpression of target genes in PANC-1 cells were confirmed (Fig. 5b and Additional file 6: Figure S5P). Colony formation (Fig. 5c), transwell experiments (Fig. 5d), and CCK-8 proliferation, cell counting assays (Fig. 5e, f) revealed that over-expressed SRSF5^250w^, but not SRSF5^250m^, promoted cell proliferation and migration of PANC-1 cells, which was reversed by additional treatments with TG003. Interestingly, overexpressed SRSF5^250w^ did not exhibit the similar capacity in BxPC-3 cells, while over-expressed SRSF5-S250P promoted proliferation and metastasis in BxPC-3 cells (Additional file 6: Figure S5A-S5E).

Considering that this difference may be caused by differential CLK1 expression, we further evaluated the functions of SRSF5 in the presence or absence of CLK1 expression. CLK1 silence significantly impaired the pro-oncogenic functions of SRSF5-WT, while overexpression of SRSF5-250P exhibited a strong ability to promote proliferation and metastasis of PANC-1 cells even after silencing of CLK1 (Fig. 5g–j). Knock down SRSF5 weaken the pro-oncogenic functions of CLK1 (Additional file 6: Figure S5F-5J). In addition, even under the condition of TG003 treatment, SRSF5-250P overexpression was still able to promote cell proliferation and metastasis, in comparison to SRSF5-WT overexpression (Additional file 6: Figure S5K-S5O). In both PANC-1 cells and BxPC-3 cells, the phosphorylation of SRSF5 seemed to be the main reason for CLK1 to regulate the EMT pathway and cell cycle pathway, as compared with the CLK1 silence alone group, SRSF5-250P overexpression caused similar changes in expressions of E-cadherin, Vimentin, p53 and p21 even in the CLK1 silenced PANC-1 cells (Fig. 5k and Additional file 6: Figure S5Q,R).

To evaluate the effect of phosphorylation of SRSF5^Ser250^ in vivo, we inoculated stable PANC-1 cells infected with control lentivirus, or CLK1-shRNA-expressing lentivirus, or stable CLK1 knockdown cell lines with overexpression of SRSF5^Ser250WT^, SRSF5^Ser250M^ or SRSF5^Ser250P^ into nude mice. Consistent with the in vitro results, in the xenograft animal model, xenografts with upregulated expression of SRSF5^Ser250P^ grew faster than other groups with upregulated expressions of SRSF5^Ser250WT^ or SRSF5^Ser250M^ after silencing of CLK1 (Fig. 5l–n). Bioluminescence imaging showed that the SRSF5^Ser250P^ group demonstrated stronger lung metastasis signal than the SRSF5^Ser250WT^ and SRSF5^Ser250M^ groups after silencing of CLK1 (Fig. 5o). Furthermore, histological analysis of the dissected lungs confirmed that the SRSF5^Ser250P^ group had more metastatic nodules than the control groups (Fig. 5p). Moreover, the SRSF5^Ser250P^ group had shorter survival time than the other two groups (Fig. 5q). Thus, our results suggest that CLK1-mediated phosphorylation of SRSF5 on Ser250 is a potential mechanism underlying the pro-oncogenic functions of CLK1 in pancreatic cancer.

### SRSF5 controlled the exon skipping of METTL14 and Cyclin L2 in pancreatic cancer cells

**Fig. 6:**
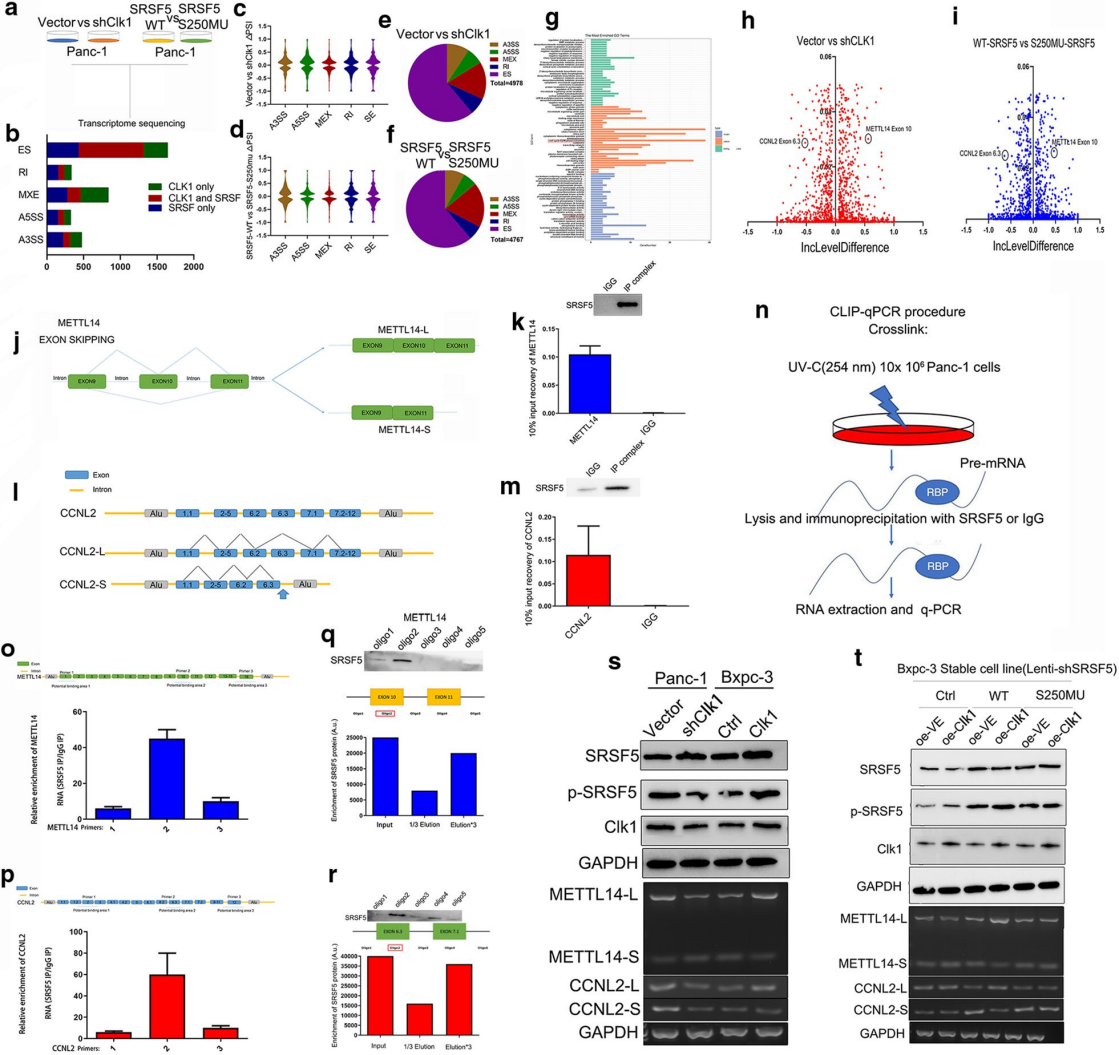
SRSF5 controlled the exon skipping of METTL14 and CyclinL2. **a** A schematic diagram shows the experimental design of transcriptome sequencing in Group A and Group B. **b** Genes with different AS patterns were represented according to the type of AS events. **c**, **d** The constituent ratios of different AS events were showed in two groups. **e**, **f** The changes of PSI of different AS events were showed in two groups. **g** GO analysis of genes with shift of exon skipping in two groups. **h**, **i** Analysis of △PSI in two groups: X axis represents IncLevelDifference: Difference expression of Exon Inclusion Isoform between Vector and sh-CLK1 (**h**) or overexpressed SRSF5wt and SRSF5mu (**i**). Y axis represents FDR. **j**, **l** Schematic diagrams show the alternative splicing of METLL14 (J) and Cyclin L2 (L) by exon skipping. **k**, **m** RIP assays were performed to confirmed the interaction between SRSF5 and pre-mRNA of METTL14 (**k**) or CyclinL2 (**m**). **n** Schematic diagrams show the CLIP-qPCR. CLIP assays were performed in Panc-1 cells to confirmed the interaction between SRSF5 and the region of METTL14 (**o**) or CyclinL2 (**p**). **q**, **r** A series of bait-oligo were constructed, and RNA pull down assays were conducted to identified the site of METTL14 (**q**), or CyclinL2 (**r**) that SRSF5 interacted with in PANC-1 cells. **s**, **t** Representative images and quantification of the relationships between CLK1-SRSF5 axis and AS patterns of METTL14 (s) and Cyclin L2 (**t**) are presented. n = 3 for each group; data are shown as mean ± SD from three independent experiments. *P < 0.05; **P < 0.01; ***P < 0.001, between the indicated groups

In order to have a global insight into the alternative splicing events regulated by CLK1-mediated SRSF5 phosphorylation on Ser250, we performed transcriptome sequencing in two groups of PC cells. Group A consisted of PANC-1 cells that were infected with control lentivirus (Lenti-CTRL) or CLK1- scramble expression lentivirus (sh-CLK1), while Group B included PANC-1 cells with stable expression of wild type SRSF5 (SRSF5^WT^) or mutated SRSF5 (SRSF250m) (Fig. 6a). The AS pattern changed in 564 genes in Group A and 659 genes in Group B (Fig. 6b). and 4978 different AS events (DAEs) in Group A were identified, 4767 different AS events in Group B were observed (Fig. 6b). The shift of PSI was showed (Fig. 6c, d), among which exon skipping was the most frequent AS event occurred in both two groups (Fig. 6e, f). Subsequent GO analysis on the genes with shift of exon skipping was performed. Results showed that the biological functions were enriched in cellular process (Fig. 6g), among which we noticed that exon 10 skipping of METTL14 got high PSI in both groups (Fig. 6h) while exon 6.3 skipping of Cyclin L2 got low PSI in both groups (Fig. 6i). Therefore, these two genes were selected to further explore their contribution to PC progression and the schematic of the alterable splicing patterns of these two genes is shown (Fig. 6j, l).

To confirm the role of CLK1 and the phosphorylation on SRSF5 250-Ser in regulating the AS patterns of METTL14 and Cyclin L2, we first performed RIP assays, and confirmed the interaction between SRSF5 and METTL14 (Fig. 6k) or Cyclin L2 (Fig. 6m). In order to identify more accurate positions of SRSF5 interacting with target genes, we designed special primers and CLIP-qPCR was performed (Fig. 6n), and the results showed that SRSF5 appeared to bind in exon10 of METTL14 and exon 6.2 of CyclinL2 (Fig. 6o, p). To further confirm the RIP and CLIP observations, we generated a series of bait-RNA to conduct RNA pulldown assays, and observed that oligo 2 of METTL14 and Cyclin L2 exhibited the strongest binding to SRSF5 (Fig. 6q, r). Furthermore, In PANC-1 and BxPC-3 cells, we found that CLK1 knockdown promoted the Cyclin L2^exon 6.3^ skipping while inhibited METTL14 ^exon 10^ skipping, whereas increased expression of CLK1 showed oppose effects (Fig. 6s). In order to provide more detailed information about the modification of CLK1-mediated exon skipping of METTL14 and Cyclin L2, we respectively transfected the vector, SRSF5 WT-expressing or SRSF5-S250Mu-expressing plasmids into BxPC-3 cells with stable silencing of SRSF5. Strikingly, we found that only in the cells which over-expression of SRSF5-WT, CLK1 was able to regulate the exon skipping of METTL14 and Cyclin L2, while CLK1 lost its regulatory effect in cells with overexpressed SRSF-250M and silencing of SRSF5 (Fig. 6t). Taken together, our data implied that the phosphorylation of SRSF5 on Ser250 by CLK1 promoted the interaction between SRSF5 and the pre-mRNA of METTL14 and Cyclin L2 and subsequent regulation of exon skipping.

### SRSF5 elevated the m6A level and promoted cell proliferation and metastasis in PC cells by inhibiting METTL14 exon skipping

**Fig. 7:**
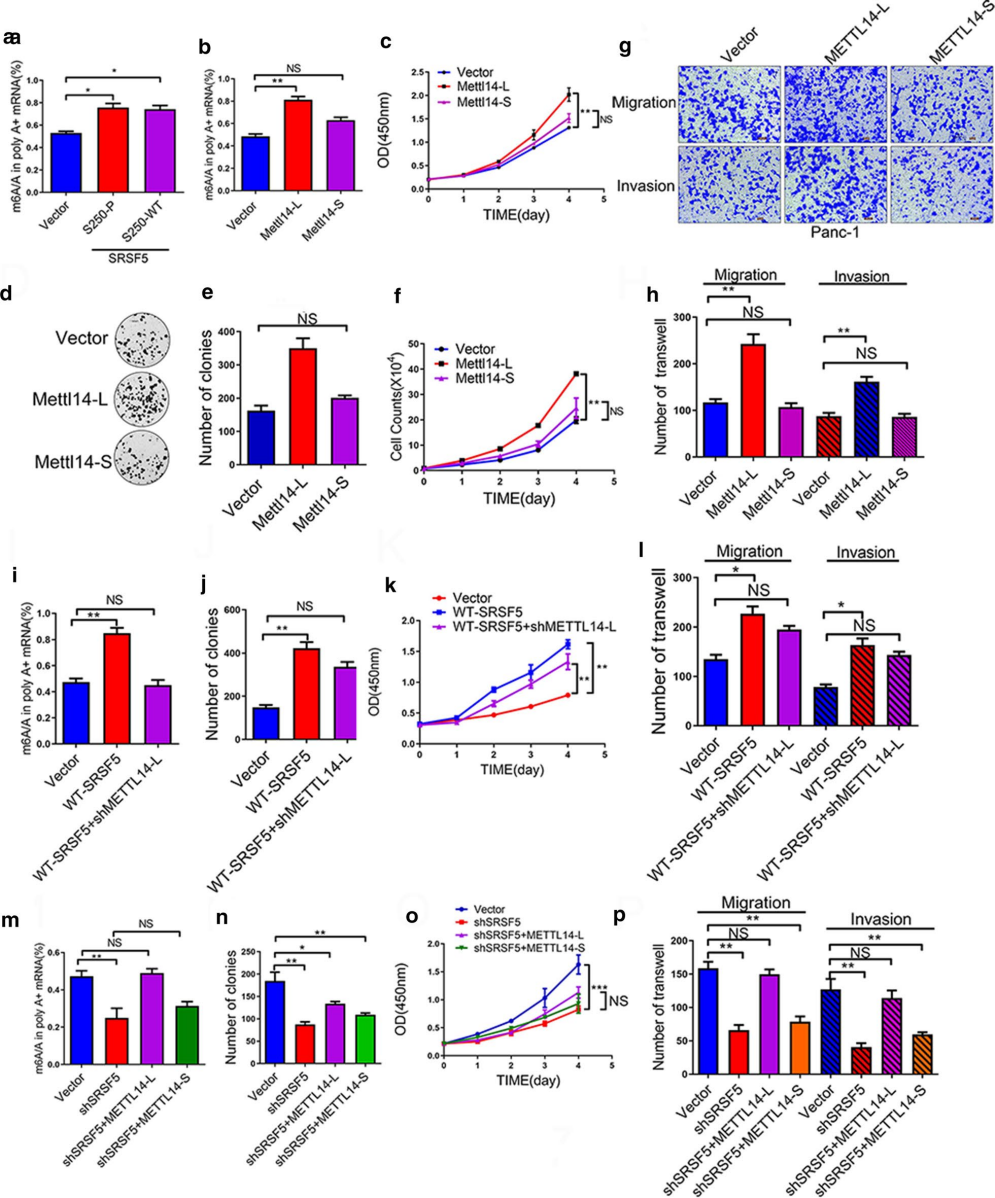
SRSF5 elevated the m6A level and promoted cell proliferation and metastasis in PC cells by inhibiting METTL14 ^exon10+^ skipping. **a**, **b** m6A immunoprecipitation assays were performed to determine the m6A levels in PANC-1 cells transduced with control vector plasmid, or plasmids expressing wild type (WT)/ phosphorylated (S250P) SRSF5 (**a**) or PANC-1 cells infected with control lentivirus, or lentivirus expressing METTL14-L or METTL14-S (**b**). **c**–**h** The proliferation ability (**c**, **f**), colony formation ability (**d**, **e**), migration and invasion ability (**g**, **h**) of PANC-1 cells infected with control lentivirus, or lentivirus expressing METTL14-L or METTL14-S were evaluated. **i**–**l** The m6A levels (**i**), colony formation ability (**j**), cell proliferation ability (**k**), migration and invasion ability (**l**) of PANC-1 cells infected with control lentivirus, or lentivirus expressing WT SRSF5 or WT SRSF5 together with METLL14 exon10-specific shRNA were evaluated. **m**–**o** The m6A levels (**m**), colony formation ability (**n**), cell proliferation ability (**o**) of PANC-1 cells infected with control lentivirus, lentivirus expressing SRSF5-specific siRNA alone, or SRSF5-specific siRNA together with METTL14-L or METTL14-S were evaluated. Representative images and quantification of the results are presented. n = 3 for each group; data are shown as mean ± SD from three independent experiments. *P < 0.05; **P < 0.01; ***P < 0.001, between the indicated groups

Considering that METTL14 is mainly involved in regulating m6A, we were curious about the influence of METTL14 alternative splicing on the m6A level. We first explored the role of the CLK1-SRSF5 axis in the regulation of m6A level in PANC-1 cells. Overexpression of SRSF5-WT and SRSF5-P, significantly increased the m6A level (Fig. 7a). We then infected PANC-1 cells with METTL14-L and METTL14-S lentivirus, and examined the m6A level, cell proliferation and metastasis. We found that METTL14-L induced an elevated m6A level in comparison to the METTL14-S and control groups (Fig. 7b). In addition, the METTL14-L group displayed more robust ability in cell proliferation (Fig. 7c, f), colony formation (Fig. 7d, e), migration and invasion (Fig. 7g, h).

In addition, SRSF5-WT overexpression (Fig. 7i–l) or CLK1 overexpression (Additional file 7: Figure S7A-S7D) caused increase in the m6A level and more active malignant behaviors including proliferation, colony formation, migration, and invasion were completely or partially reversed by silencing of METTL14-L. Moreover, the impairments in these malignant behaviors of PANC-1 cells caused by SRSF5 silencing (Fig. 7m–p), or CLK1 silencing (Additional file 7: Figure S7E-S7H), or treatments with TG003 (Additional file 7: Figure S7I-S7L), were completely or partially reversed by ectopic expression of METTL14-L, but not METTL14-S. Taken together, the elevated m6A level and pro-oncogenic roles of the CLK1-SRSF5 axis in pancreatic cancer cells are dependent on inhibiting the METTL14^△exon10+^ skipping.

### SRSF5 promoted tumor proliferation in PC cells by promoting cyclinL2^△exon6.3^ skipping

**Fig. 8:**
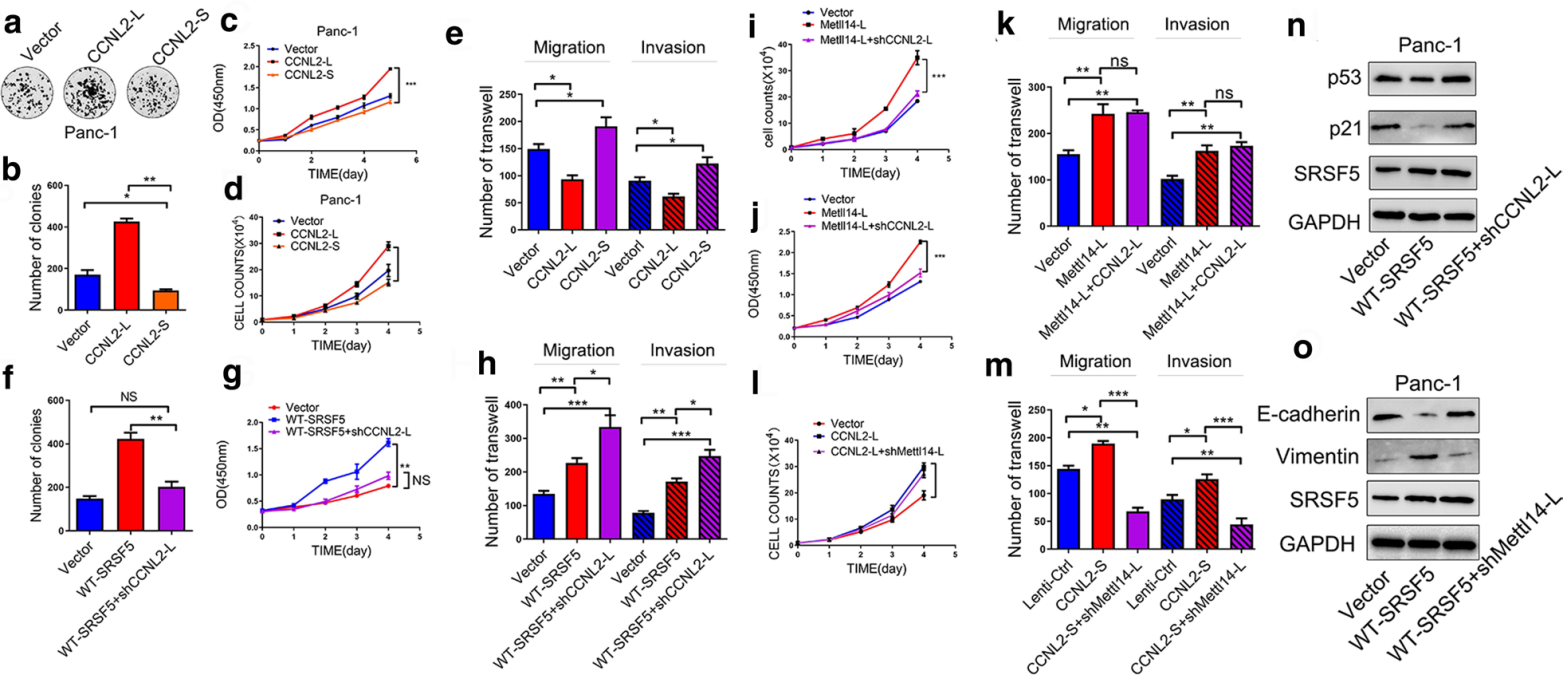
SRSF5 promoted tumor proliferation in PC cells by promoting cyclinL2^△exon6.3^ skipping. **a**–**e** PANC-1 cells were infected with control lentivirus, or lentivirus expressing cyclinL2^△exon6.3- (CCNL2-L)^ or cyclinL2^△exon6.3+ (CCNL2-S)^. The colony formation ability (**a**, **b**), cell proliferation ability (**c**, **d**), and migration and invasion ability (**e**) of the indicated stable cells were evaluated. **f**–**h** PANC-1 cells were infected with control lentivirus, or lentivirus expressing WT SRSF5, or lentivirus expressing WT SRSF5 and CCNL12-L-specific siRNA. The colony formation ability (**f**), cell proliferation ability (**g**), and migration and invasion ability (**h**) of the indicated stable cells were evaluated. **i**–**k** PANC-1 cells were infected with control lentivirus, or lentivirus expressing METTL14-L, or lentivirus expressing METTL14-S and CCNL12-L-specific shRNA. The cell proliferation ability (**i**, **j**), and migration and invasion ability (**k**) of the indicated stable cells were evaluated. **l**, **m** PANC-1 cells were infected with control lentivirus, or lentivirus expressing CCNL12-L, or lentivirus expressing CCNL12-L and METTL14-L-specific siRNA. The cell proliferation ability (**l**), and migration and invasion ability (**m**) of the indicated stable cells were evaluated. **n**, **o** The protein levels of p21, p53 (**n**), E-cadherin, N-cadherin, and Vimentin (**o**) in PANC-1 cells as described in (**f**–**h**) were determined by western blot assays. Representative images and quantification of the results are presented. n = 3 for each group; data are shown as mean ± SD from three independent experiments. *P < 0.05; **P < 0.01; ***P < 0.001, between the indicated groups

Cyclin L2 was reported to regulate cell cycle and proliferation in many types of cancers [33, 34]. To explore the impacts of AS on the functions of cyclinL2 in PC cells, we generated CyclinL2^△exon6.3+^ (CCNL2-S) and CyclinL2^△exon6.3−^ (CCNL2-L) lentivirus, and evaluated how they could change the malignant behaviors of PANC-1 cells. Compared with the control cells, overexpressed CCNL2-L promoted colony formation and cell proliferation, while overexpressed CCNL2-L inhibited the proliferation (Fig. 8a–d). Notably, we also found that CCNL2-S promoted migration and invasion of PDAC cells, while CCNL2-L suppressed the invasive migration (Fig. 8e). We also evaluated the contributions of CyclinL2^△exon6.3^ skipping in the CLK1-SRSF5 axis-mediated regulation of PC malignant behaviors. Considering that SRSF5 promoted CyclinL2^△exon6.3^ skipping, we introduced sh-CCNL2-L in PANC-1 cells with stable SRSF5 overexpression. Compared with the SRSF5 overexpressed cells, additional knockdown of CCNL2-L significantly reduced colony formation (Fig. 8f), suppressed cell proliferation (Fig. 8g), and increased cell migration and invasion (Fig. 8h). These changes were accompanied with the alterations on the protein levels of cell cycle regulation associated molecules p21 and p53 as well as EMT markers E-cadherin and Vimentin (Fig. 8n, o and Additional file 8: Figure S8M).

Moreover, in PANC-1 cells with SRSF5 silence (Additional file 8: Figure S8A-S8C), or CLK1 silence (Additional file 8: Figure S8D-S8F), or TG003 treatments (Additional file 8: Figure S8J-S8L), further overexpression of CCNL2-L markedly restored cell proliferation, but had opposite effect on migration and invasion of PANC-1 cells. In addition, CCNL2-L silence erased the promotive effects of CLK1 overexpression on cell proliferation, but exhibited opposite effect on metastasis of PANC-1 cells (Additional file 8: Figure S8G-S8I).

Furthermore, we investigated whether there were functional reciprocal interactions between these two alternative splicing events. We found that CCNL2-L reversed the increased proliferation but did not reversed the enhanced metastasis that were caused by overexpression of METTL14-L in PANC-1 cells (Fig. 8i–k). On the other hand, silence of METTL14-L reversed the enhanced metastasis, while did not reverse the increased proliferation caused by overexpression of CCNL2-S (Fig. 8l, m). Therefore, we speculated that the pro-proliferative role of the CLK1-SRSF5 axis was at least partially dependent on CyclinL2^△exon6.3^ skipping, which strongly promoted proliferation while inhibited metastasis of PC cells.

### Aberrant alternative splicing of METTL14^△exon10^ and CyclinL2^△exon6.3^ contributed to the clinical prognosis of PDAC

**Fig. 9:**
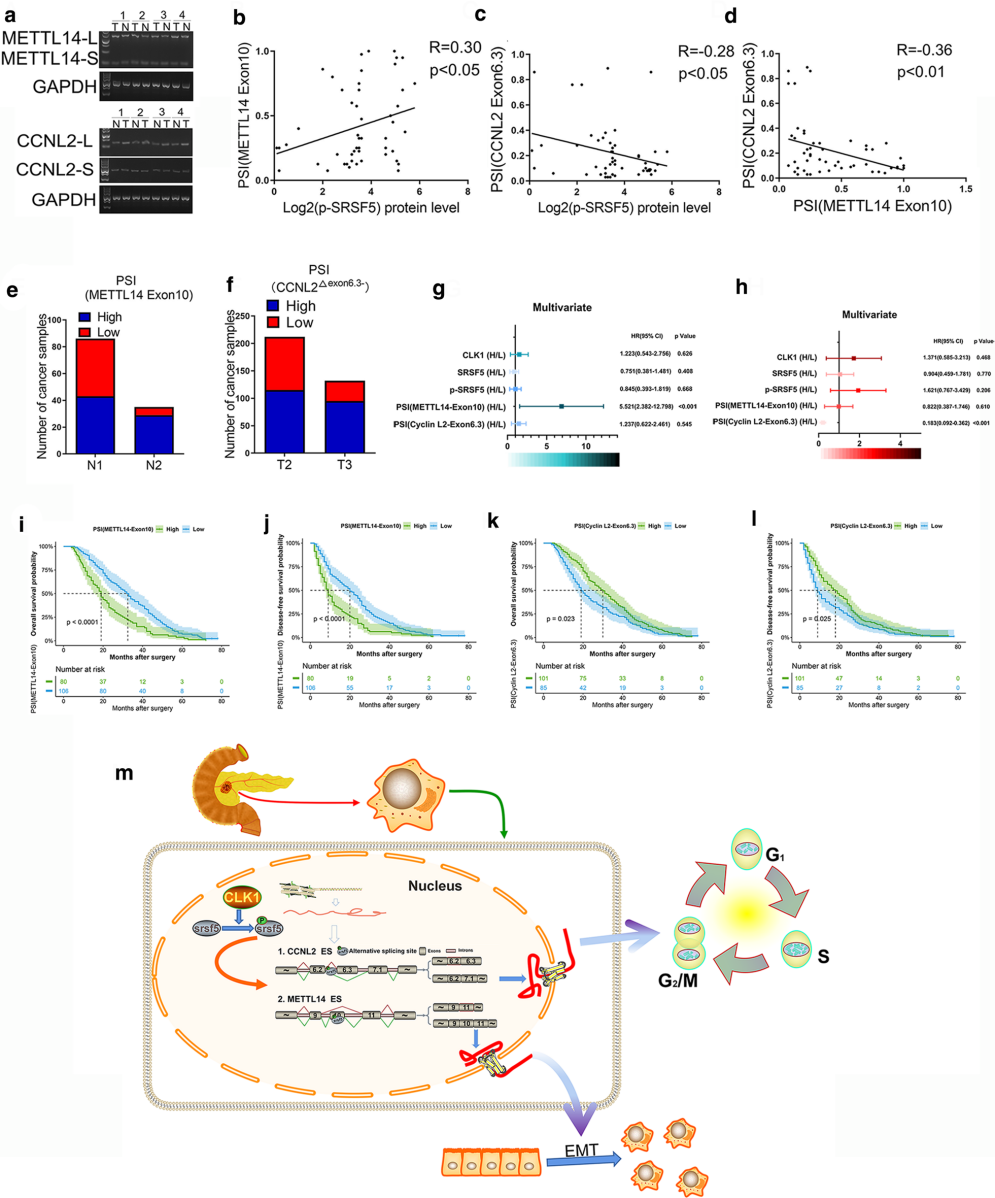
Aberrant alternative splicing of METTL14^△exon10^ and CyclinL2^△exon6.3^ possessed prognostic values for PDAC patients. **a** PCR of cyclinL2^△exon6.3^ and METTL14^△exon10^ in PDAC samples was evaluated by PCR. Representative images of DNA gel electrophoresis are shown. **b**–**d** The correlations between the levels of METTL14^exon10^ (**b**) or CyclinL2^△exon6.3^ (**c**) and the expression levels of p-SRSF5 and their correlations (**d**) were determined by IHC staining in tumor tissues were performed. **e**, **f** The correlations between the levels of METTL14^exon10^ (**e**) or CyclinL2^△exon6.3^ (**f**) and lymphatic metastasis and tumor size. **g**, **h** Multiple-factor analysis between CLK1-SRSF5-METTL14/SRSF5 axis and lymphatic metastasis and tumor size. **I**–**l** Overall and disease-free survival analyses were performed to assess the impact of PSI of METTL14^exon10^ or CyclinL2^△exon6.3^ shift in PDAC patients. **m** A proposed working model of the CLK1-SRSF5 axis induced aberrant exon skipping of m6A methyltransferase METTL14 and Cyclin L2 in promoting the development of the pancreatic cancer through regulating cell cycle progression, cell proliferation, and metastasis. Overall (**a**, **c**) and disease-free (**b**, **d**) survival analyses were performed to assess the impact of PSI of METTL14^exon10^ or CyclinL2^△exon6.3^ shift in PDAC patients in external corhort

In order to further clarify the relationship between alternative splicing of METTL14/Cyclin L2 and the clinical prognosis of pancreatic cancer patients, we performed PCR and evaluated the percent spliced in index (PSI) of METTL14^△exon10^ and CyclinL2^△exon6.3^ in 52 pairs PDAC samples. Results showed that the PSI of METTL14^△exon10^ elevated while the PSI of CyclinL2^△exon6.3^ reduced in PDAC tumor samples (Fig. 9a). We also evaluated the relationship between the expression of P-SRSF5 and the PSI shift of METTL14 and Cyclin L2 in the clinical samples, and found that the expression of P-SRSF5 was positively correlated with the occurrence of METTL14^△exon10^ (Fig. 9b) and negatively correlated with the occurrence of CyclinL2^△exon6.3^ (Fig. 9c). Moreover, PSI of METTL14^△exon10^ was negatively correlated with the PSI of CyclinL2^△exon6.3^ (Fig. 9d).

Patients with high PSI of METTL14^△exon10^ have more lymph node metastases than those with low PSI of METTL14^△exon10^ (Fig. 9e), On the other hand, patients with low PSI of CyclinL2^△exon6.3^ have larger tumors than those with high PSI of CyclinL2^△exon6.3^(Fig. 9f). Multivariable analysis confirmed that high PSI of METTL14^△exon10^ was positive related to lymphatic metastasis, while high PSI of CyclinL2^△exon6.3^ was positive related negatively correlated tumor sizes. The overall survival rate (Fig. 9i, k, Additional file 9: S9A,C) rate and disease-free survival rate (Fig. 9j, l, Additional file 9: S9B,D), for patients with high PSI of METTL14^△exon10^ or low PSI of CyclinL2^△exon6.3^ was significantly lower than that for patients with low PSI of METTL14^△exon10^ or high PSI of CyclinL2^△exon6.3^. Therefore, aberrant alternative splicing of METTL14 and CyclinL2 had potential prognostic values for PDAC patients.

## Discussion

Though numerous therapeutic strategies have been developed to improve the treatment outcomes of PC, it is still a devastating disease with a complex and not clearly elucidated physiopathology. It is urgent to explore the pathogenesis and search for new biomarkers of PC. In this study, we first demonstrated that human PDAC tissues and cells had higher levels of CLK1 than the matched normal tissues, and higher CLK1 expression predicted worse prognosis of PDAC patients. Moreover, we first revealed that SRSF5 acted as important phosphorylation target protein of CLK1 by label-free quantitative phosphorylation proteomics. Most importantly, we firstly observed global AS events regulated by the CLK1-SRSF5 axis via transcriptome sequencing, and identified the shift of AS patterns in METTL14 and Cyclin L2 as core regulatory events. We also deeply explore the functions of different transcripts of METTL14 and Cyclin L2 (CCNL2), and their complex interactions. We characterized splicing changes and associated m6A modification. We firstly demonstrated that the accumulation of CLK1-SRSF5 axis increased splicing towards METTL14 to accelerate the protein level of METTL14^exon10+^, which was closely related to m6A modification and invasion in PC cells. Meanwhile, the CLK1-SRSF5 axis also promoted PC cell proliferation via increased alternative splicing of Cyclin L2. Combined with our clinical data, there was a significant correlation among CLK1, SRSF5, METTL14 and CCNL2 expression in clinical pancreatic tissues, further supporting the regulatory axis of CLK1, SRSF5, METTL14 and CCNL2 (Fig. 9m). To the best of our knowledge, the present findings establish the first link between RNA splicing machinery and the writer of m6A modification. Importantly, we also showed that abnormal splicing also affected the transcripts of cell cycle-related proteins (p21 and p53) and the writer of m6A modification, and this process is essential during pancreatic carcinogenesis.

Accumulating evidence has confirmed that alternative splicing plays an important role in the development of PDAC, including EMT, inflammatory responses, and resistance to herapies [10, 35, 36]. CLK1 was a dual specificity kinase which was known to phosphorylate splicing factors like SR proteins and to regulate their distribution and functions within the nucleoplasm [31, 32, 37, 38]. Aberrant phosphorylation of splicing factors by CLK1 has been demonstrated to be critically involved in disorders of AS events in human diseases including cancer [39]. However, the role of CLK1 and targeted SR proteins in PC has not been intensively elucidated. Here, we identified that the expression of CLK1 was elevated in PDAC by high-through RNA sequencing and data mining of public datasets. We further explored the functions of CLK1 in different PDAC cell lines, and showed that CLK1 acted as a multifunctional protein and promoted PDAC growth and metastasis both in vitro and in vivo.

Previous studies have reported that CLK1 was elevated in the late S and early G2 phase [35]. In our study, knockdown of CLK1 resulted in a G2/M phase arrest, meanwhile the expressions of p53 and p21 were increased, which may explain why CLK1 promoted the PDAC proliferation while its knockdown exhibited opposite effects. To further explore the functions and underlying mechanisms, we performed the GO and KEGG analyses in DEGs, and confirmed that CLK1 acted as an important cell cycle regulator. CLK1 was reported to regulated alternative splicing via phosphorylating many types of SR proteins [32, 38–40], while the major target SR protein remains unknown in PDAC. Thus, we performed label-free quantitative phosphorylation proteomics assays. The results showed the major proteins phosphorylated by CLK1 were related to mRNA splicing, which is consistent with previous studies [32, 38–40]. Among them, we identified the phosphorylation of SRSF5 on serine 250 was strongly affected by CLK1 expression. For the first time, we confirmed the important role of the CLK1-SRSF5 axis in PDAC developments in vivo and in vitro. We found that in the BxPC-3 cells with a low CLK1 expression, enhanced p-SRSF expression promoted the malignant behaviors of cells while overexpression of WT-SRSF5 did not shown such effects. On the other hand, in PANC-1 cells with down-regulated CLK1 expression, enhanced p-SRSF expression can rescue the suppressive ability of CLK1 down-regulation, while enhanced WT-SRSF5 expression did not. Consistent with the data of in vitro experiment, our clinical data also demonstrated that the clinical value of SRSF5 relied on CLK1 protein expression. Collectively, our data suggest that CLK1 controls the PDAC malignant behaviors via regulating the phosphorylation status of SRSF5 but not its expression.

SR proteins are widely involved in the regulation of malignant tumor-related AS events, and it controls the inclusion or skipping of specific exons through interacting with one specific recognition sequence in the target pre-mRNA [36, 41, 42]. In our study, transcriptome sequencing was preformed to explore the AS events regulated by the CLK1-SRSF5 axis, from which we found that the major AS events were related to m6A modification and cell cycle regulation. Among them, we noticed that the PSI of exon 10 of METTL14 and exon 6.3 of Cyclin L2 were strongly impacted by the CLK1-SRSF5 axis. Moreover, RIP, CLIP-qPCR, RNA-pulldown assays proved that METTL14 and Cyclin L2 are the new target of SRSF5. These interactions between SRSF5 and pre-mRNAs resulted in acceleration of the skipping of exon 6.3 of CCNL2 mRNA, and decrease of the skipping of exon 10 of METTL14 mRNA. Therefore, SRSF5 might be required for the constitutive splicing of METTL14 pre-mRNA to ensure the inclusion of exon 10 in the correct order, by preventing exon skipping through interaction with exon 10 of the METTL14 mRNA. On the other hand, SRSF5 binding with the exon 6.3 of Cyclin L2 can promote the exon 6.3 skipping. These results together indicate that SRSF5 plays different roles in regulating AS events of different molecules.

Cyclin L2 is a member of the cyclin family, and it has been reported to be involved in the regulation of cell cycle progression and in the signal transduction of the apoptosis pathway [33, 43]. Zhuo et.al have revealed that Cyclin L2 expression was upregulated in ventricular septum tissue of ventricular septal defect (VSD) patients, and demonstrated that Cyclin L2 can regulate growth, differentiation, and apoptosis of P19 cells via inhibiting the WNT pathway [34]. In our study, the functional differences of enhanced and suppressed Cyclin L2^exon6.3^ skipping were firstly identified. While Cyclin L2^exon6.3^ skipping promoted proliferation and inhibited metastasis, inhibition of Cyclin L2^exon6.3^ skipping promoted metastasis and inhibited proliferation of PC cells. Although our results support the functions of Cyclin L2^exon6.3^ in regulating cell growth regardless of cell types, the detailed mechanisms of Cyclin L2^exon6.3^ in regulating cell metastasis of PC cells remain to be elucidated in future.

Apart from AS, m6A modifications also plays a pivotal role in mRNA post-translation modification [44]. Dysregulated m6A participates in the initiation and progression of many human diseases, including cancers [19, 45–49]. For example, through alteration of RNA translation efficiency and RNA stability, m6A dysregulation affects the signaling pathways of some key tumor suppressors and oncogenes in multiple types of cancers. METTL14 is the key methyltransferase responsible for m6A modifications, and has been demonstrated to inhibit the metastasis of hepatocellular carcinoma through m6A-dependent primary microRNA processing events [50]. In pancreatic cancer, upregulation of METTL14 expression was reported to lead the reduction of PERP levels via m6A modification, thus promoting the metastasis of PC [24]. Therefore, we were curious about whether the shift of AS could result in this phenotypic difference. In this study, we showed for the first time that the CLK1-SRSF5 axis can regulate the METTL14^exon10^ skipping in PC cells, and the METTL14-L is more important than METTL14-S in regulating the level of m6A modification. In addition, we found that the stimulatory effect of activation of the CLK1-SRSF5 axis on PC cell migration and invasion can be rescued by the enhanced METTL14^△exon10^ skipping. These data revealed the importance of exploring for the roles of AS in the writer of m6A modification. Moreover, this may help explain why the CLK1-SRSF5 axis promoted Cyclin L2^exon6.3^ skipping and still promoted metastasis of PC cells.

## Conclusion

In summary, for the first time, we systematically evaluated the role of the CLK1-SRSF5 axis in the development of pancreatic cancer, as well as the prognosis values of this pathway. Mechanistically, we demonstrated that the CLK1-SRSF5 axis promoted pancreatic cancer proliferation via promoting Cyclin L2^exon 6.3^ skipping, and enhanced the ability of cell migration and invasion through inhibiting METTL14^exon 10^ skipping. Notably, we are the first to demonstrate the critical role of the CLK1-SRSF5 axis in regulating m6A methylation in pancreatic cancer via impacting on the alternative splicing pattern of METTL14^△exon10^. Moreover, we are also the first to report the shift of alternative splicing pattern in m6A modification related proteins around the world. Our next focus is to identify the target gene responsible for the shift of METTL14^△exon10^ skipping. Our study sheds new light on targeting the CLK1-SRSF5 axis and m6A regulators to develop effective therapeutics for patients with pancreatic cancer.

## Supplementary Information

**Additional file 1: Figure S1.** Higher CLK1 expression in tumors correlated with worse prognosis of PDAC patients. (**A**) The expression of CLK1 in pancreatic tumor and adjacent normal tissues from TCGA database were analyzed. (T = 179, N = 171). (**B**) The expression of CLK1 in 156 paraffin-embedded specimens from the external cohort was determined by TMA-based IHC staining. Representative IHC images are shown. (**C**)The relative CLK1 staining intensity was scored. (**D–H**) Kaplan–Meier analyses of the correlations between CLK1 expression and overall survival (**D**), disease-free survival (**E**) of all PDAC patients, or Stage II patients (**F**, **I**), or Stage III patients (**G**, **H**) in the internal cohort. *P < 0.05; **P < 0.01; ***P < 0.001, between the indicated groups.

**Additional file 2: Figure S2.** The impacts of CLK1 overexpression and knockdown on the proliferation of 8988 cells. (**A**, **F**) 8988 cells with stable CLK1 overexpression or Knockdown were confirmed by western blot. Cell proliferation (**B**, **C**, **G**, **H**), colony formation (**D**, **I**), and cell cycle progression (**E**, **J**) in the indicated cell lines were evaluated. n = 3 for each group; (**K**) The expression of CLK1, p53 and p21 in the tissues from the xenograft tumor were determined by TMA-based IHC staining. Representative IHC images are shown. (**L**-**M**) The expressions of p53 and p21 were negatively correlated with the expression of CLK1 in the xenograft tumors. Data are shown as mean ± SD from three independent experiments. *P < 0.05; **P < 0.01; ***P < 0.001, between the indicated groups.

**Additional file 3: Figure S3.** The impacts of CLK1 overexpression and knockdown on the migration and invasion of 8988 cells. (**A**-**H**) 8988 cells with stable CLK1 overexpression (**A**-**D**) and with CLK1 knockdown (**E**–**H**) were generated. Cell migration and invasion ability (**A**-**C**, **E**–**G**), and wound healing ability (**D**, **H**) in the indicated cell lines were evaluated. n = 3 for each group; data are shown as mean ± SD from three independent experiments. *P < 0.05; **P < 0.01; ***P < 0.001, between the indicated groups.

**Additional file 4: Figure S4I**. CLK1 and SRSF5 had prognostic values for PDAC patients. (**A**) The quantification of the expression level of p-SRSF5 in different PC cell lines. (**B**) The quantification of the results of Fig. 4L. (**C**) The quantification of the results of Fig. 4M. (**D**, **E**) The phosphorylation level of SRSF5 on Ser250 rely on the expression of CLK1. (**B**-**J**) Kaplan–Meier analyses of the correlations between CLK1/SRSF5 expression and overall survival or disease-free survival of PDAC patients.

**Additional file 5: Figure S4II.** Interaction between CLK1 and SRSF5 in PANC-1 cells, BxPC-3 cells, and 293T cells. (**A**-**E**) HA-tagged CLK1 and Flag-tagged SRSF5 were transfected into PANC-1 cells (**A**), or BxPC-3 cells (**B**-**C**), or 293T cells (**D**-**3**). Co-immunoprecipitation and western blot assays were performed with the indicated antibodies.

**Additional file 6: Figure S5.** CLK1-mediated SRSF5 phosphorylation on Ser250 contributed to the malignant behaviors of pancreatic cancer cells. (**A**-**E**) BxPC-3 cells were infected with control lentivirus, or lentivirus expressing wild type (WT)/mutated (S250MU)/phosphorylated (S250P) SRSF5. The cell proliferation ability (**A**, **B**), colony formation ability (**C**), and migration and invasion ability (**D**, **E**) of the indicated stable cells were evaluated. (**F**-**J**) PANC-1 cells were infected with control lentivirus or lentivirus overexpressing CLK1 or lentivirus overexpressing CLK1 together with shSRSF5. The cell proliferation ability (**F**, **G**), colony formation ability (**H**), and migration and invasion ability (**I**, **J**).The stable cells were infected with control lentivirus, or lentivirus expressing wild type (WT)/mutated (S250MU)/phosphorylated (S250P) SRSF5 were simultaneous treatment with TG003 (**K**–**O**)were evaluated. The cell proliferation ability (**K**, **L**), colony formation ability (**M**), and migration and invasion ability (**N**, **O**). (**P**)The quantification of the results of Fig. 5B. (**Q**, **R**)The quantification of the results of Fig. 5K. n = 3 for each group; data are shown as mean ± SD from three independent experiments.*P < 0.05; **P < 0.01; ***P < 0.001, between the indicated groups.

**Additional file 7: Figure S7.** METTL14 exon skipping functioned downstream of the CLK1 signaling to control the malignant behaviors of pancreatic cancer cells. (**A**-**D**) PANC-1 cells were infected with control lentivirus, or lentivirus expressing CLK-1 alone or CLK-1 together with shRNA specific to METTL14exon10+. The m6A level (**A**), colony-formation ability (**B**), cell proliferation ability (**C**), and migration and invasion ability (**D**) of the indicated stable cells were evaluated. (**E**–**H**) PANC-1 cells were infected with control lentivirus, or lentivirus expressing CLK-1-specific shRNA or CLK-1-specific shRNA together with METTL14exon10+. The m6A level (**E**), colony-formation ability (**F**), cell proliferation ability (**G**), and migration and invasion ability (**H**) of the indicated stable cells were evaluated. (**I**-**L**) PANC-1 cells were treated with TG003 alone or together lentivirus infection-mediated overexpression of METTL14-L or METTL14-S. The m6A level (**I**), colony-formation ability (**J**), cell proliferation ability (**K**), and migration and invasion ability (**L**) of the indicated stable cells were evaluated. n = 3 for each group; data are shown as mean ± SD for three independent experiments. *P < 0.05; **P < 0.01; ***P < 0.001, between the indicated groups.

**Additional file 8: Figure S8.** CyclinL2^△exon6.3^ skipping functioned downstream of the CLK1 signaling to control the malignant behaviors of pancreatic cancer cells. (**A**-**C**) PANC-1 cells were infected with control lentivirus, or lentivirus expressing shRNA-specific to SRSF5 alone or together with overexpression of CCNL2-L or CCNL2-S. The cell proliferation ability (**A**), colony-formation ability (**B**), and migration and invasion ability (**C**) of the indicated stable cells were evaluated. (**D**-**F**) PANC-1 cells were infected with control lentivirus, or lentivirus expressing shRNA-specific to CLK1 alone or together with overexpression of CCNL2-L or CCNL2-S. The cell proliferation ability (**D**), colony-formation ability (**E**), and migration and invasion ability (**F**) of the indicated stable cells were evaluated. (**G**-**I**) PANC-1 cells were infected with control lentivirus, or lentivirus expressing CLK1 alone or CLK1 together with lentivirus expressing shRNA-specific to CCNL2-L. The cell proliferation ability (**G**), colony-formation ability (**H**), and migration and invasion ability (**I**) of the indicated stable cells were evaluated. (**J**-**L**) PANC-1 cells were treated with TG003 alone or together with lentivirus infection-mediated overexpression of CCNL2-L or CCNL2-S. The cell proliferation ability (**J**), colony-formation ability (**K**), and migration and invasion ability (**L**) of the indicated stable cells were evaluated. (**M**) The quantification of the results of Fig. 8n, o. n = 3 for each group; data are shown as mean ± SD from three independent experiments. *P < 0.05; **P < 0.01; ***P < 0.001, between the indicated groups.

**Additional file 9: Figure S9.** Aberrant alternative splicing of METTL14^△exon10^ and CyclinL2^△exon6.3^ possessed prognostic values for PDAC patients in external corhort.

## Data Availability

All data in our study are available upon request.

## Abbreviations

PDAC: Pancreatic ductal adenocarcinoma
FBS: Fetal bovine serum
GEO: Gene Expression Omnibus
HR: Hazard ratio
CCK-8: Cell counting kit 8
IHC: Immunohistochemistry
OS: Overall survival
DFS: Disease free survival
PDAC: Pancreatic ductal carcinoma
TCGA: The Cancer Genome Atlas
CLK1: Cdc2-like kinases SRSFs: SR-splicing factors
METTL14: Methyltransferase-like 14
AS: Alternative splicing
