# Supplementary figures and images for: CLK1/SRSF5 pathway induces aberrant exon skipping of METTL14 and Cyclin L2 and promotes growth and metastasis of pancreatic cancer
Source: J Hematol Oncol. 2021 Apr 13;14:60. doi: 10.1186/s13045-021-01072-8 (PMC8045197; doi:10.1186/s13045-021-01072-8)

A

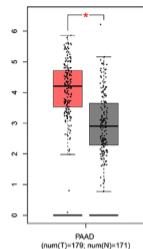

B

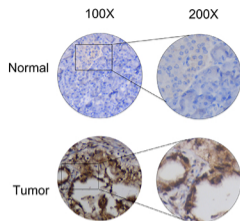

D

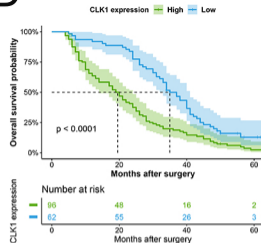

F

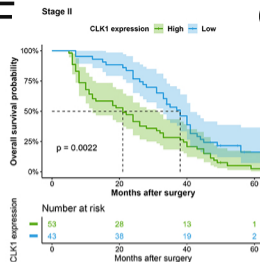

G

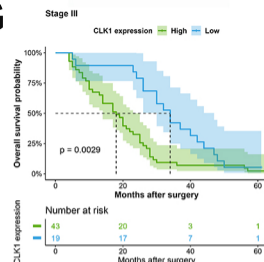

E

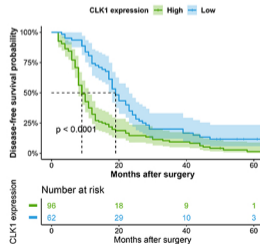

H

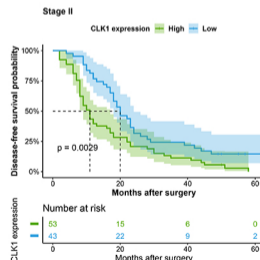

I

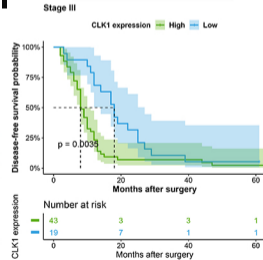

C

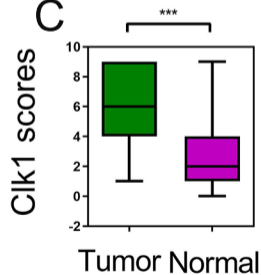

Supplemental Figure 1

Supplement: Supplementary file 1 — Additional file 1: Figure S1. Higher CLK1 expression in tumors correlated with worse prognosis of PDAC patients. (A) The expression of CLK1 in pancreatic tumor and adjacent normal tissues from TCGA database were analyzed. (T = 179, N = 171). (B) The expression of CLK1 in 156 paraffin-embedded specimens from the external cohort was determined by TMA-based IHC staining. Representative IHC images are shown. (C)The relative CLK1 staining intensity was scored. (D–H) Kaplan–Meier analyses of the correlations between CLK1 expression and overall survival (D), disease-free survival (E) of all PDAC patients, or Stage II patients (F, I), or Stage III patients (G, H) in the internal cohort. *P < 0.05; **P < 0.01; ***P < 0.001, between the indicated groups. [file 13045_2021_1072_MOESM1_ESM.pdf]

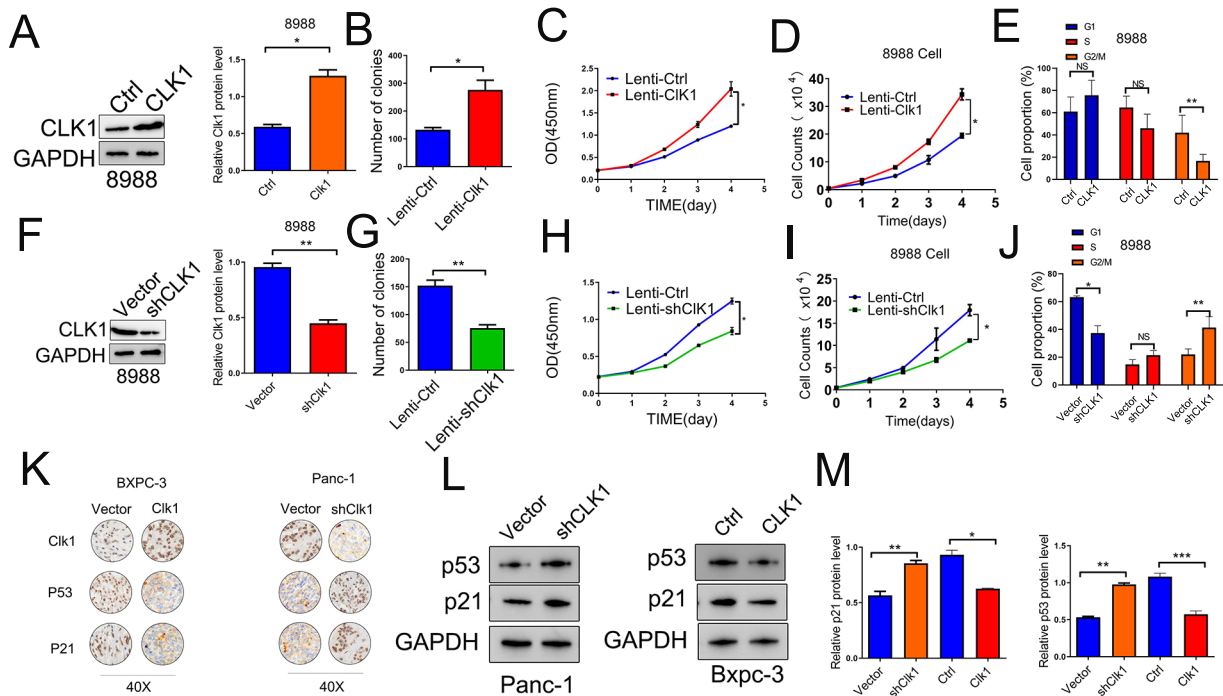

Supplemental Figure 2

Supplement: Supplementary file 2 — Additional file 2: Figure S2. The impacts of CLK1 overexpression and knockdown on the proliferation of 8988 cells. (A, F) 8988 cells with stable CLK1 overexpression or Knockdown were confirmed by western blot. Cell proliferation (B, C, G, H), colony formation (D, I), and cell cycle progression (E, J) in the indicated cell lines were evaluated. n = 3 for each group; (K) The expression of CLK1, p53 and p21 in the tissues from the xenograft tumor were determined by TMA-based IHC staining. Representative IHC images are shown. (L-M) The expressions of p53 and p21 were negatively correlated with the expression of CLK1 in the xenograft tumors. Data are shown as mean ± SD from three independent experiments. *P < 0.05; **P < 0.01; ***P < 0.001, between the indicated groups. [file 13045_2021_1072_MOESM2_ESM.pdf]

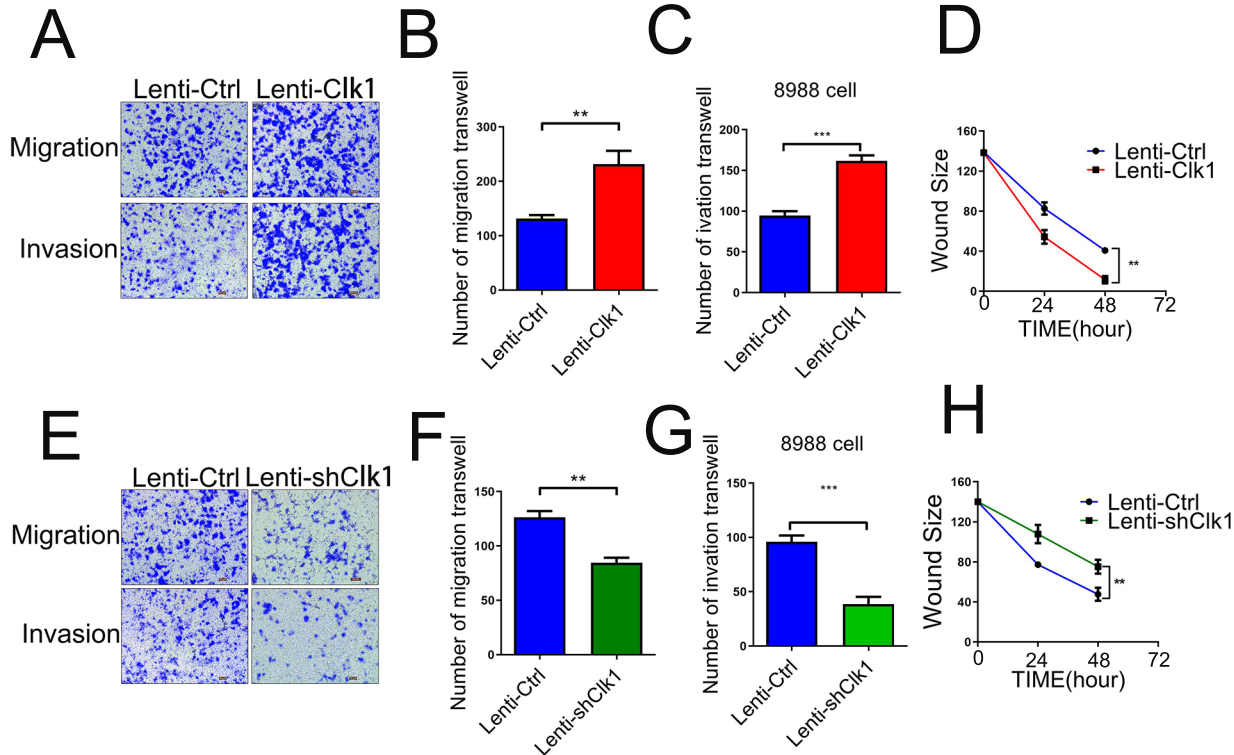

Supplemental Figure 3

Supplement: Supplementary file 3 — Additional file 3: Figure S3. The impacts of CLK1 overexpression and knockdown on the migration and invasion of 8988 cells. (A-H) 8988 cells with stable CLK1 overexpression (A-D) and with CLK1 knockdown (E–H) were generated. Cell migration and invasion ability (A-C, E–G), and wound healing ability (D, H) in the indicated cell lines were evaluated. n = 3 for each group; data are shown as mean ± SD from three independent experiments. *P < 0.05; **P < 0.01; ***P < 0.001, between the indicated groups. [file 13045_2021_1072_MOESM3_ESM.pdf]

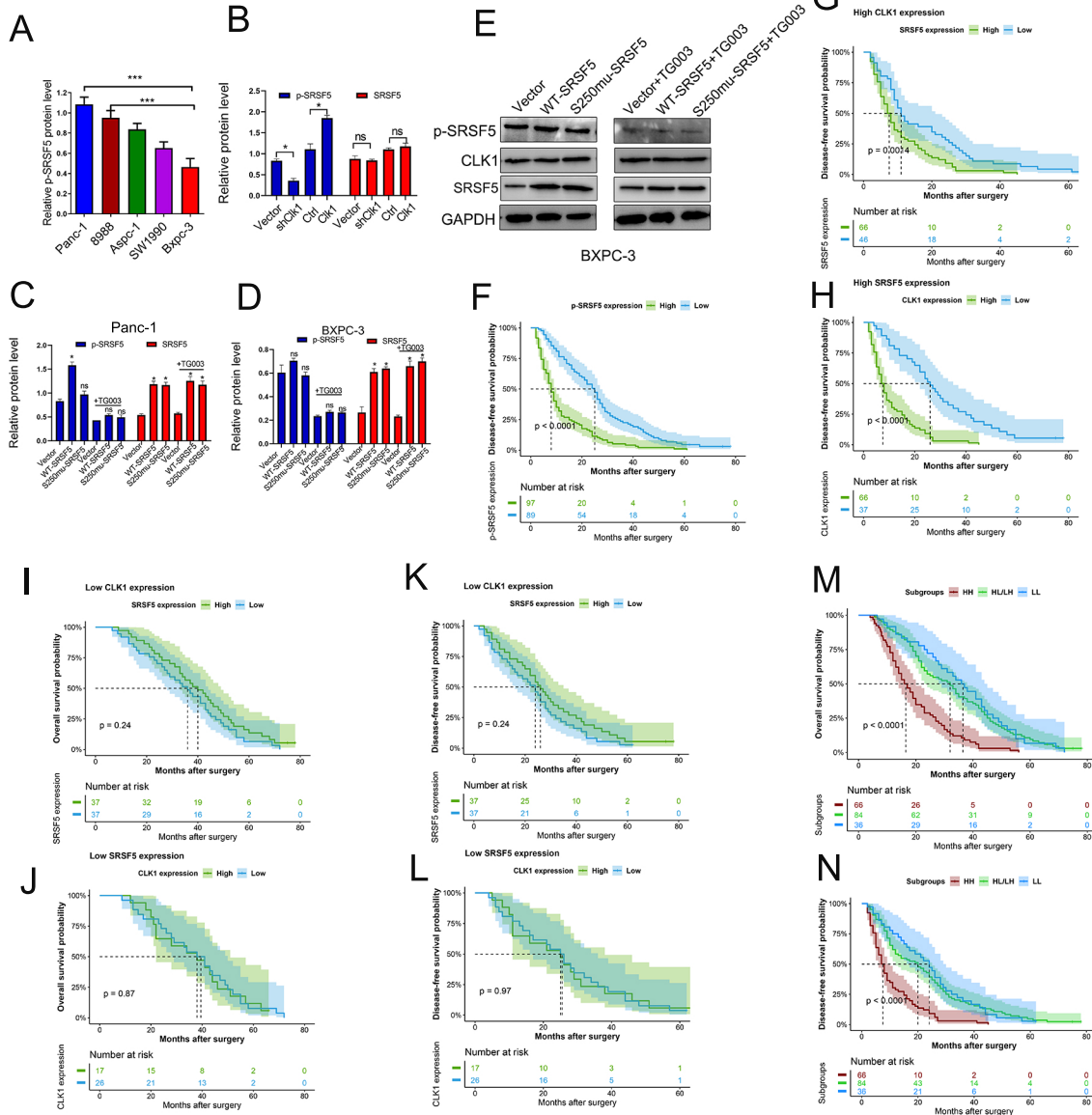

Supplemental Figure 4I

Supplement: Supplementary file 4 — Additional file 4: Figure S4I. CLK1 and SRSF5 had prognostic values for PDAC patients. (A) The quantification of the expression level of p-SRSF5 in different PC cell lines. (B) The quantification of the results of Fig. 4L. (C) The quantification of the results of Fig. 4M. (D, E) The phosphorylation level of SRSF5 on Ser250 rely on the expression of CLK1. (B-J) Kaplan–Meier analyses of the correlations between CLK1/SRSF5 expression and overall survival or disease-free survival of PDAC patients. [file 13045_2021_1072_MOESM4_ESM.pdf]

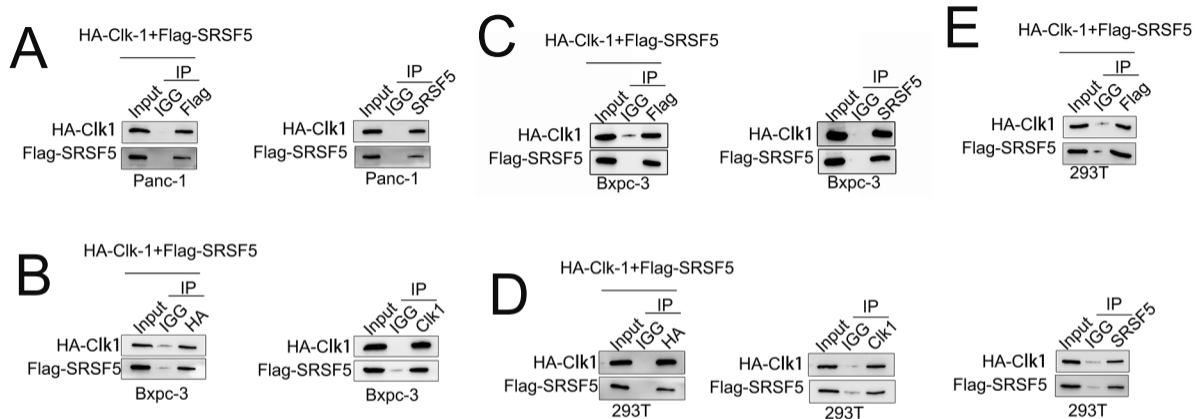

Supplemental Figure 4II

Supplement: Supplementary file 5 — Additional file 5: Figure S4II. Interaction between CLK1 and SRSF5 in PANC-1 cells, BxPC-3 cells, and 293T cells. (A-E) HA-tagged CLK1 and Flag-tagged SRSF5 were transfected into PANC-1 cells (A), or BxPC-3 cells (B-C), or 293T cells (D-3). Co-immunoprecipitation and western blot assays were performed with the indicated antibodies. [file 13045_2021_1072_MOESM5_ESM.pdf]

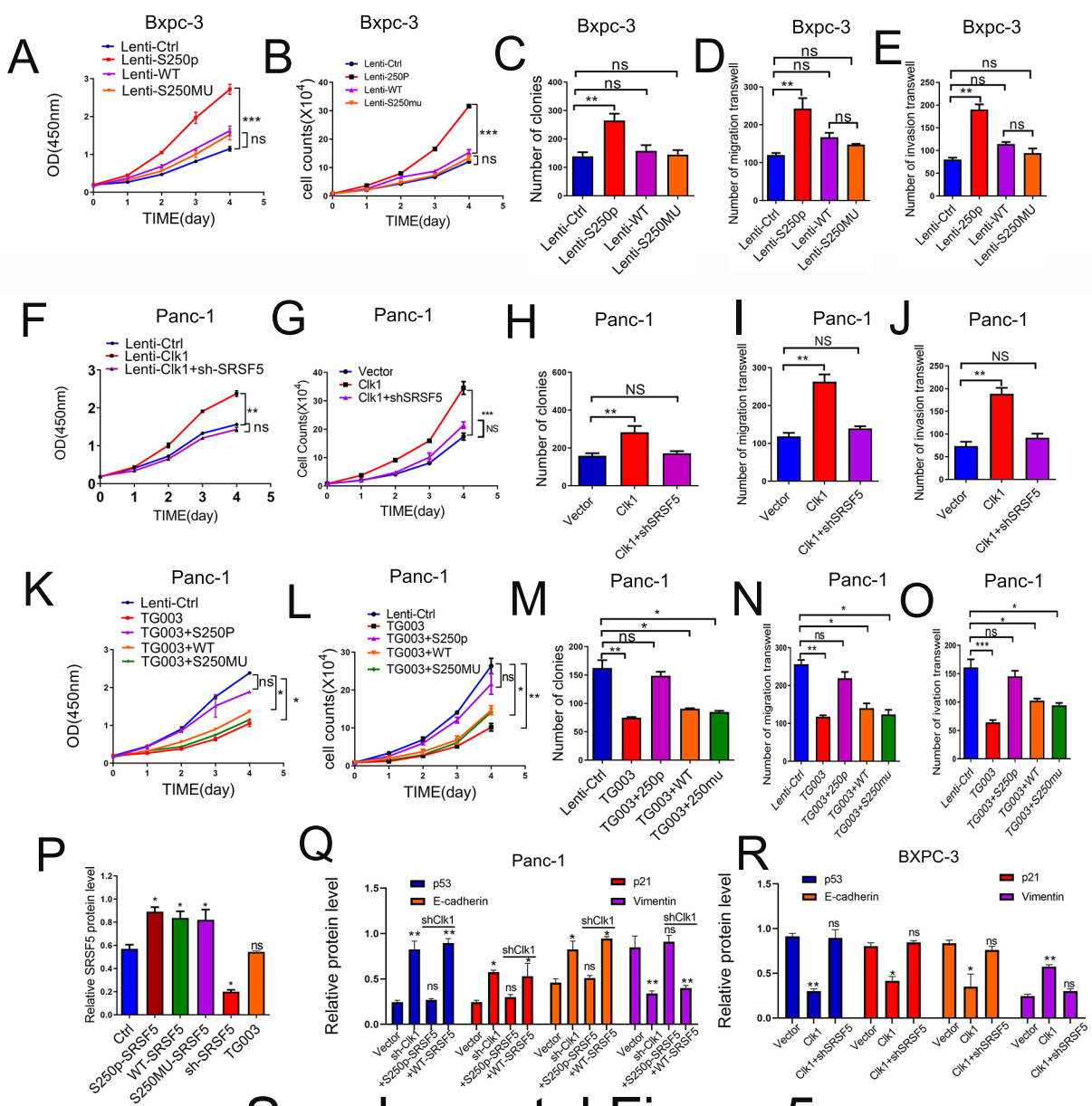

Supplemental Figure 5

Supplement: Supplementary file 6 — Additional file 6: Figure S5. CLK1-mediated SRSF5 phosphorylation on Ser250 contributed to the malignant behaviors of pancreatic cancer cells. (A-E) BxPC-3 cells were infected with control lentivirus, or lentivirus expressing wild type (WT)/mutated (S250MU)/phosphorylated (S250P) SRSF5. The cell proliferation ability (A, B), colony formation ability (C), and migration and invasion ability (D, E) of the indicated stable cells were evaluated. (F-J) PANC-1 cells were infected with control lentivirus or lentivirus overexpressing CLK1 or lentivirus overexpressing CLK1 together with shSRSF5. The cell proliferation ability (F, G), colony formation ability (H), and migration and invasion ability (I, J).The stable cells were infected with control lentivirus, or lentivirus expressing wild type (WT)/mutated (S250MU)/phosphorylated (S250P) SRSF5 were simultaneous treatment with TG003 (K–O)were evaluated. The cell proliferation ability (K, L), colony formation ability (M), and migration and invasion ability (N, O). (P)The quantification of the results of Fig. 5B. (Q, R)The quantification of the results of Fig. 5K. n = 3 for each group; data are shown as mean ± SD from three independent experiments.*P < 0.05; **P < 0.01; ***P < 0.001, between the indicated groups. [file 13045_2021_1072_MOESM6_ESM.pdf]

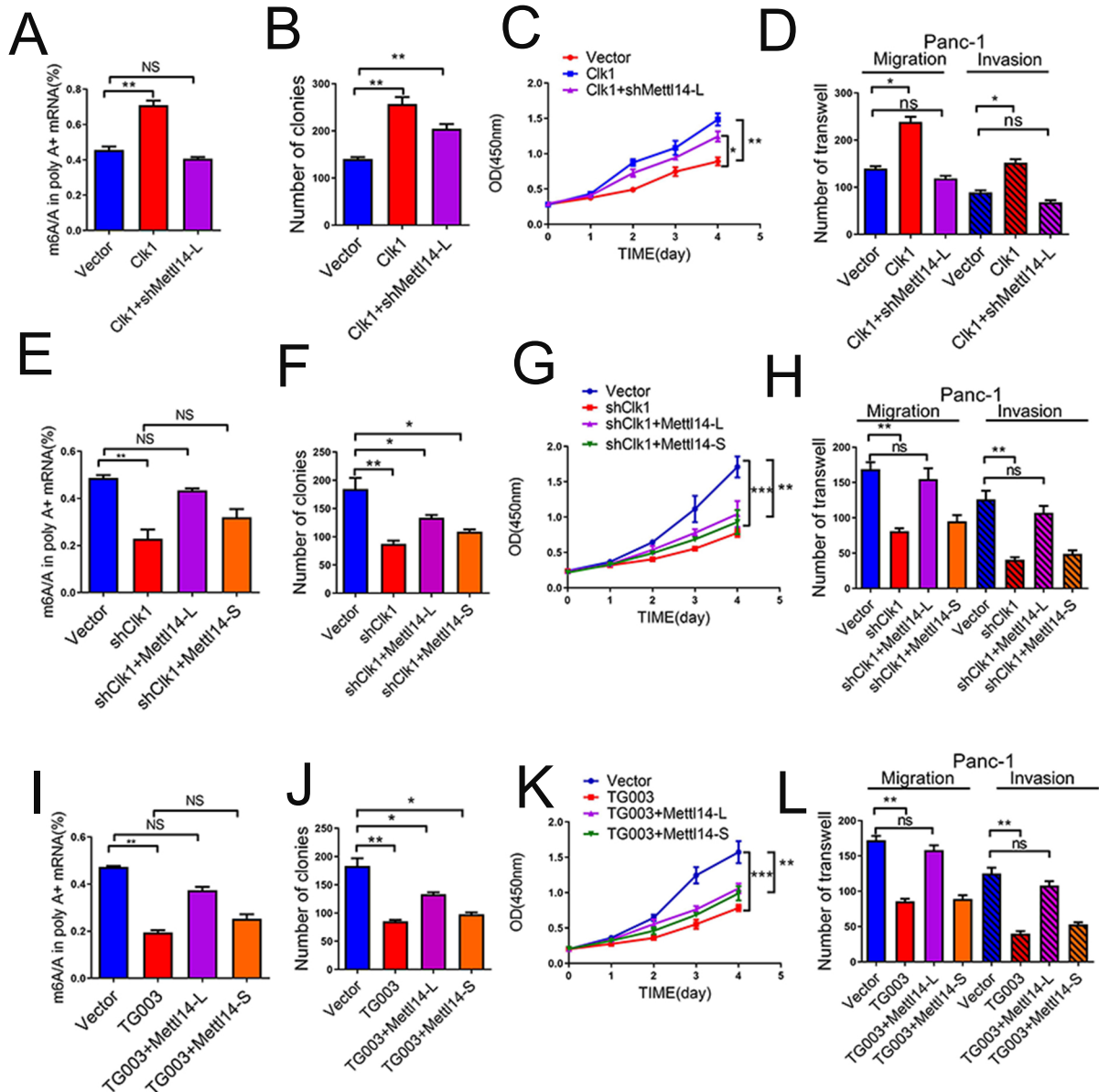

Supplemental Figure 7

Supplement: Supplementary file 7 — Additional file 7: Figure S7. METTL14 exon skipping functioned downstream of the CLK1 signaling to control the malignant behaviors of pancreatic cancer cells. (A-D) PANC-1 cells were infected with control lentivirus, or lentivirus expressing CLK-1 alone or CLK-1 together with shRNA specific to METTL14exon10+. The m6A level (A), colony-formation ability (B), cell proliferation ability (C), and migration and invasion ability (D) of the indicated stable cells were evaluated. (E–H) PANC-1 cells were infected with control lentivirus, or lentivirus expressing CLK-1-specific shRNA or CLK-1-specific shRNA together with METTL14exon10+. The m6A level (E), colony-formation ability (F), cell proliferation ability (G), and migration and invasion ability (H) of the indicated stable cells were evaluated. (I-L) PANC-1 cells were treated with TG003 alone or together lentivirus infection-mediated overexpression of METTL14-L or METTL14-S. The m6A level (I), colony-formation ability (J), cell proliferation ability (K), and migration and invasion ability (L) of the indicated stable cells were evaluated. n = 3 for each group; data are shown as mean ± SD for three independent experiments. *P < 0.05; **P < 0.01; ***P < 0.001, between the indicated groups. [file 13045_2021_1072_MOESM7_ESM.pdf]

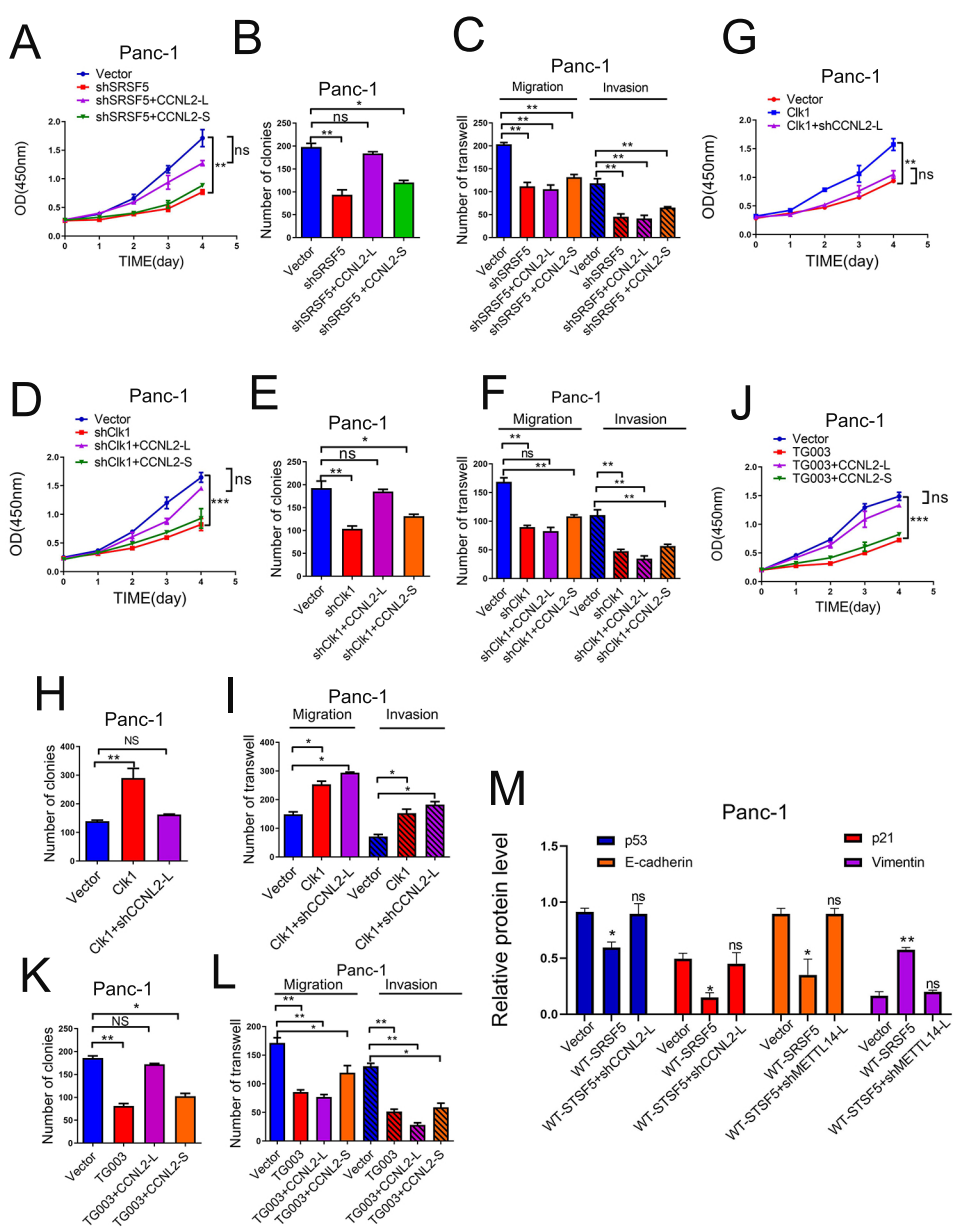

Supplemental Figure 8

Supplement: Supplementary file 8 — Additional file 8: Figure S8. CyclinL2△exon6.3 skipping functioned downstream of the CLK1 signaling to control the malignant behaviors of pancreatic cancer cells. (A-C) PANC-1 cells were infected with control lentivirus, or lentivirus expressing shRNA-specific to SRSF5 alone or together with overexpression of CCNL2-L or CCNL2-S. The cell proliferation ability (A), colony-formation ability (B), and migration and invasion ability (C) of the indicated stable cells were evaluated. (D-F) PANC-1 cells were infected with control lentivirus, or lentivirus expressing shRNA-specific to CLK1 alone or together with overexpression of CCNL2-L or CCNL2-S. The cell proliferation ability (D), colony-formation ability (E), and migration and invasion ability (F) of the indicated stable cells were evaluated. (G-I) PANC-1 cells were infected with control lentivirus, or lentivirus expressing CLK1 alone or CLK1 together with lentivirus expressing shRNA-specific to CCNL2-L. The cell proliferation ability (G), colony-formation ability (H), and migration and invasion ability (I) of the indicated stable cells were evaluated. (J-L) PANC-1 cells were treated with TG003 alone or together with lentivirus infection-mediated overexpression of CCNL2-L or CCNL2-S. The cell proliferation ability (J), colony-formation ability (K), and migration and invasion ability (L) of the indicated stable cells were evaluated. (M) The quantification of the results of Fig. 8n, o. n = 3 for each group; data are shown as mean ± SD from three independent experiments. *P < 0.05; **P < 0.01; ***P < 0.001, between the indicated groups. [file 13045_2021_1072_MOESM8_ESM.pdf]

A

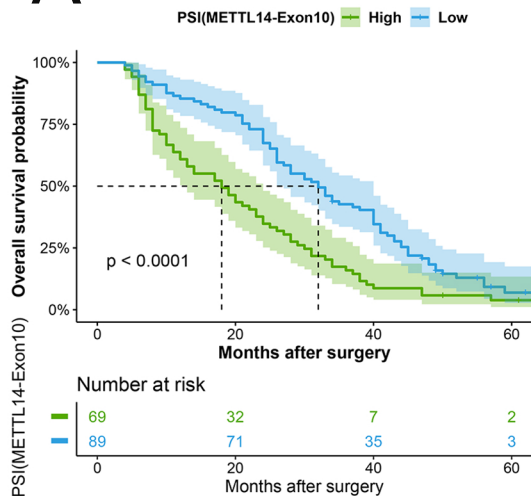

C

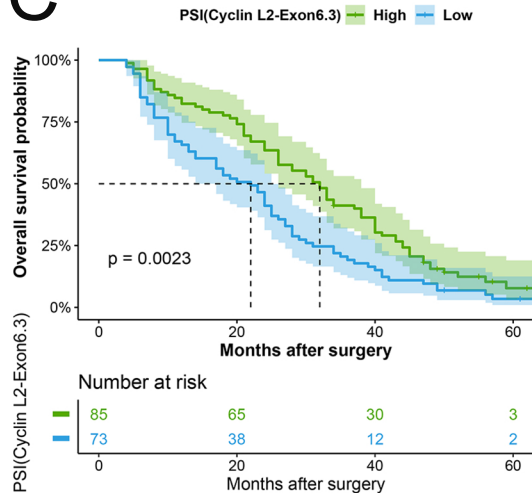

B

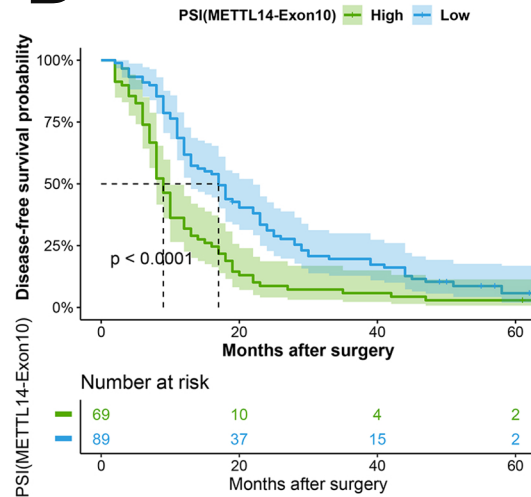

D

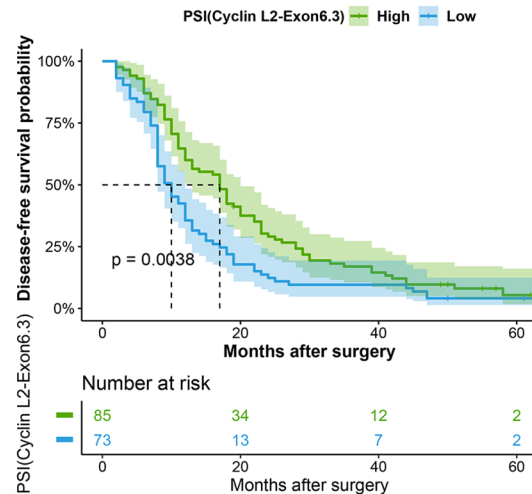

# Supplemental Figure 9

Supplement: Supplementary file 9 — Additional file 9: Figure S9. Aberrant alternative splicing of METTL14△exon10 and CyclinL2△exon6.3 possessed prognostic values for PDAC patients in external corhort. [file 13045_2021_1072_MOESM9_ESM.pdf]
